# The Impact of Particulate Matters and Nanoparticles on Thermoplastic Polymer Coatings and Paint Layers

**DOI:** 10.3390/polym14122477

**Published:** 2022-06-17

**Authors:** Pierre-Antoine Héritier

**Affiliations:** Atelier HERITIER SàRL, Route des Acacias 45 b, Les Acacias, 1227 Genève, Switzerland; pamfheritier@bluewin.ch

**Keywords:** nanoparticles, ultra-fine particles (UFPs), dust, dirt, acrylic polymers, painted artwork

## Abstract

This article attempts to highlight a phenomenon that more or less permanently damages emulsion paint layers, the surfaces of which remain sufficiently permeable for dust particles to become permanently anchored there; when the particles are nanometric, this can cause a permanent change in appearance. Based on scientific documents, empirical observations, laboratory analyses, case studies, and reconstructions of characteristic pictorial layers, this paper aims to highlight the medium- and long-term risks that alter these surfaces, in order to realize strategies for better prevention. The physico-chemical nature of these vulnerable materials will be discussed first, followed by the dust’s involvement; finally, the topic will be illustrated through concrete examples, with photos taken using digital, 4 K optical, and Scanning Electron Microscope equipment (SEM), in order to show how the problem of dust particle accumulation impacts even the most contemporary works of art.

## 1. Introduction

This study was motivated by the observation, over the last fifteen years, of a demonstrable increase in difficulties regarding the surface cleaning of emulsion paint layers, whatever the techniques used; investigations into the cleaning of paint layers [1,2,3,4,5] led to further understanding of some of these phenomena, involving the irreversibility of certain types of soiling on acrylic paint layers. 

**Figure 1:**
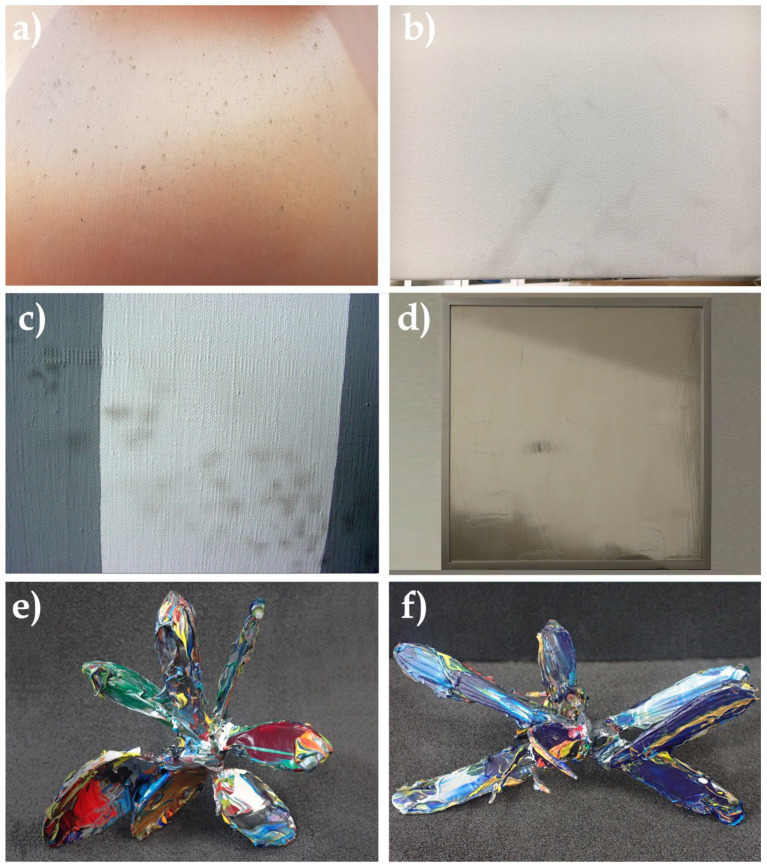
(**a**) Detail from an artwork by Allan Mc Collum, *Three Perfect Vehicles*, 1988/2004, 203.2 cm × 91.4 cm × 91.4 cm, made from acrylic latex paint on glass fiber. The artwork is exhibited outside and has a thin layer of particulate matter on its surface. (**b**) Detail of a work in acrylic paint on canvas, dating from 1998: in the shape of a very extended halo, particulate matter and UFPs are deeply anchored; this damage seems almost irreversible. (**c**) Detail of a work in acrylic paint on canvas dating from 1975: the particulate matter is deeply anchored and has clearly been deposited by bubble-wrap packing via direct contact with the surface. (**d**) Bertrand Lavier, *Untitled* (from the *Mirror* series—clear medium on an acrylic mirror, contained in the original artist’s frame), 61 cm × 61 cm. (**e**) Lucas Samaras, work without title (*Utensils* #49), from the 2001 series *Utensils*, acrylic on metal, 12 cm × 12 cm × 14 cm. (**f**) Lucas Samaras, work without title (*Utensils* #73), from the 2001 series *Utensils*, acrylic on metal, 21 cm × 12 cm × 8 cm.

Whether in the form of painted sculptures or paintings (Figure 1), the type of superficial soiling most frequently encountered is mainly composed of dust deposited on the surface of the pictorial layer, although this becomes more and more tenacious and difficult to remove mechanically (by dusting) or during cleaning when in the aqueous phase.

Unavoidable dust, whether in museums or in a private environment, is well known as an issue in the conservation world [6,7,8,9,10,11,12,13,14,15,16,17,18,19,20]; however, environmental activity is changing and both people and objects seem, unfortunately, to face new forms of pollution that increasingly impact public health, as well as affecting the conservation of works of art due to the ever-increasing spread of nanoparticles in the atmosphere, both outdoors and indoors.

This type of very fine deposit is also typical of the fine dust aggregates found on packaging films that are placed in direct contact with the surfaces of works to be packaged: the particles are released by the transfer of charges and by mechanical contact with the pictorial surfaces, which trap them by progressive harpooning; this is accentuated, among other things, by electrostatic interactions (see below).

These trapping phenomena are facilitated by the soft nature of the substrate and by movement due to the relatively low glass transition temperature (Tg) [21] characteristic of acrylic paint layers.

This insidious pollution, which sometimes seriously affects the conservation of works of art, increasingly concerns fine particles and nanoparticles.

## 2. Acrylic/Vinyl Layers: Characteristics and Vulnerabilities

Acrylic and vinyl paints are made from thermoplastic polymers, which vary in softness depending on the ambient temperature.

This property gives them great elasticity, which is highly appreciated, but they can sometimes stiffen when the temperature drops; conversely, the risks linked to the slight residual stickiness of the paint layers are very real when the glass transition temperature (Tg) of the polymer is greater than 70 °C (ibid. [21]) This includes basically all paint layers and polychrome coatings that were of interest in this study.

### 2.1. Drying, Composition, and Temperature Influence

The formation of a film by coalescence depends on the complex interactions of several factors, such as the nature of the polymer particles and the chemical structure of the surfactant molecules. Its quality depends on the size and distribution of the particles, which influence their compaction, the efficiency of compaction, and the coalescence of the particles favored by polymers with a low glass transition temperature (Tg) [22].

Thermoplastic polymers soften and become more fluid when heated. However, after cooling, they regain their initial properties. The action of heat results in a simple physical transformation that does not alter their chemical composition or their linear covalent structure (ibid. [5]). It is important to specify the complexity of the composition of the emulsions, integrating, among other materials, pigments and fillers (extenders), as well as surfactants, deformers, biocides and fungicides, plasticizers, dispersion agents such as protective colloids, and pH correctors/stabilizers or coalescing agents in the form of solvents, such as hexylene glycol, which favors a better assembly and interpenetration of the grains of the emulsion (ibid. [21]), these items are never completely freely available. Furthermore, the process of implementation by the artist is mostly completely unknown to us, such as the consideration or not of the MFT (minimum film-forming temperature) or the impact of an accidental electrolyte on the dilution of the paint or other mediums, or of drying inhibiter.

### 2.2. Triboelectricity and Surfactants

The dielectric constant and its role in the dusting of a paint layer must also be taken into account: this depends on the presence of dipoles, which may come from polar groups in the macromolecule or from dissymmetrical molecules that are present in the same way as pigments and plasticizers. These triboelectricity phenomena are fundamentally dependent on the ambient temperature and humidity (ibid. [21]) (see below).

Consider once again that, in a paint layer or acrylic coating, significant amounts of non-ionic surfactants are contained in the paint surface. It is, therefore, important to note the influence of the ambient temperature on the surfactants contained in these coatings and the acrylic paint layers [23,24,25].

When the RH rises (above 35% RH), the surfactants tend to penetrate the paint layer, dragging inside it all the surface dirt, among other things.

When the RH drops (below 35% RH), the surfactants tend to penetrate the paint layer, causing dirt and various products to surface. These phenomena, which have a tendency to hysteresis, ultimately trap dirt in the paint layer of the work, fouling it at depth (ibid. [23,24,25]).

### 2.3. Vulnerabilities

Nevertheless, in the modern era, awareness has grown regarding the relevant vulnerabilities, which are now much more widely analyzed in the medical and environmental fields.

Many authors emphasize the need for advanced scientific methods to demonstrate the possible impact of soiling on works of art (ibid. [22,25,26,27,28,29,30]), as well as the damage caused by materials commonly found indoors, such as VOCs (ibid. [22]) and PAHs (see below).

Generally, acrylic paint layers are rarely varnished, which makes them all the more sensitive to the dust and dirt that can be deposited directly on them and penetrate the surface (ibid. [20]) As has been shown, the very nature of acrylic paints, where the surface remains relatively soft at room temperature, as well as their physicochemical nature, offer an ideal trap for various surrounding dirt particles, the anchorage of which can prove extremely problematic, making certain operations and cleaning procedures particularly hazardous or even damaging (ibid. [20]).

## 3. Nanoparticles: Generalities

### 3.1. Production and Transmission

Nanoparticles are sometimes part of highly technical products used in medicines, as well as in paints and coatings (banned in food since 2021), in packaging and in cosmetics, and can also be disseminated accidentally. The average size of a virus is from 10 to 70 nm; in daily life, titanium dioxide, with a particle size of between 3 and 15 nm, is very often present, for example in sun creams.

However, according to current studies, it is mainly the “anthropogenic” sources of pollution (i.e., created by human activity) that are the biggest producers of particles, even if there are also some natural, terrigenous particles emitted by erosion, volcanic activities, forest fires, marine activities (salt aerosols), and organic compounds (gas/particle transformations) [31].

### 3.2. Air Pollution

It is necessary to state here that nanoparticles and particles of soot are not a new phenomenon: in the past, their unintentional production and their inconveniences were already reported, and historical ordinances to limit their production have been pronounced. For instance, an edict of Charles VI, dated 1382, prohibits the emission of “foull smelling and nauseating smoke” in Paris and Rouen [32]. Nevertheless, in the modern era, nanoparticles have become more significant in both the sanitary and environmental fields.

In the 2000s, governments heavily focused on the topic of thin particles with sizes of 10 to 2.5 microns (PM 10, PM 2.5) (ibid. [31]) and tried to implement European and American directives for better air quality. However, since 2016, there has been a decrease in the quantity of these particles in urban atmospheres (except when close to traffic); although, unfortunately, an increase in thin particle deposits on artistic paints was observed in our own field. These particle deposits, as shown below, are nanometric in nature.

Currently, the governments of numerous countries are beginning to legislate regarding particulate matter (PM) of 0.5 (particles sized 0.5 microns). Caused by human activity and airborne in nature, these particles are more concentrated at high temperatures and in high solar radiation because of nucleation behavior and the chemical reactions furthered under those environmental conditions [33,34,35,36,37,38,39,40,41,42,43,44,45,46,47,48,49,50,51].

During winter, UFPs are much more significant because of increased domestic and commercial heating and car emissions, but these have only recently been identified.

**Figure 2:**
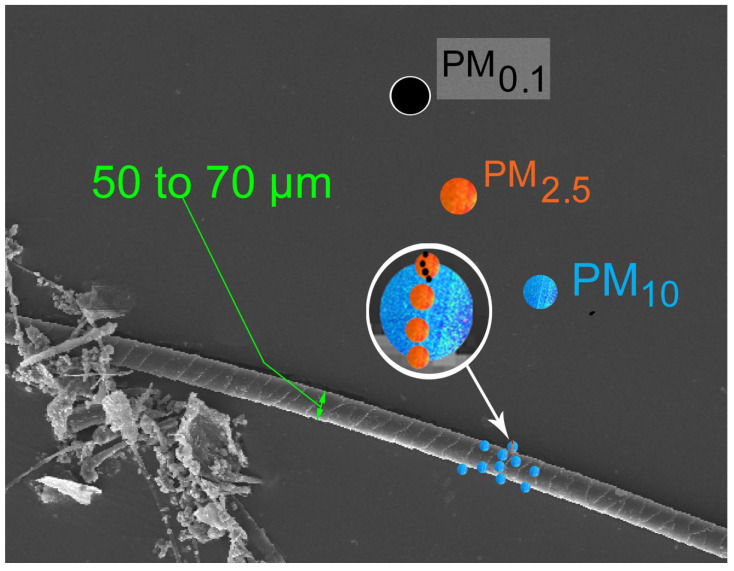
Zoom in a SEM image (magnification 150×). In order to demonstrate the size of these nanoparticles (one billionth (10^9^) of a meter), this presents a comparison with a single hair: the diameter is nearly 80,000 nm (a red blood cell measures 7000 nm). This picture of a hair taken with an SEM allows us to locate nanoparticles (PM 0.1) among other elements of PM 10 and PM 2.5. Their sizes are between the size of an atom and that of a virus or bacterium.

Although a level of PM 0.1 is not normally taken into account when assessing air quality, there is a paradox to be identified, the consequences of which are closely related to our own observations. The governmental directives taken against certain sizes of particles (Figure 2) have actually increased the production of UFPs: this signifies that UFPs are outside the norm, although they are particularly penetrating, and their impact on and inside paint layers might be dramatic because they are hard to remove, or because they are already and definitively trapped inside (ibid. [33,34,35,36,37,38,39,40,41,42,43,44,45,46,47,48,49,50,51]).

### 3.3. Impacts

In the field of the restoration of works of art, this type of airborne deposit on polychrome painted or sculpted surfaces can increasingly be observed. Often blackish in color, these deposits differ from the dirt that has to be commonly sanitized from the surfaces of works of art, such as coarse dust, organic deposits, and mineral stains.

Studies and investigations devoted to this emerging pollution problem have allowed us to envisage and better comprehend a process that is difficult to interpret, but that is very real: the virtual impossibility of eliminating traces on many pictorial layers. These minute traces of dust are equivalent in size to the pigments, with very fine particles that can be deposited on the face of an artwork and that will penetrate the micrometric pores of a pictorial surface more easily while taking advantage of the viscoelastic nature of the binder (ibid. [21]).

Their mobility and shape, therefore, allow them to penetrate more deeply into the pictorial surfaces, helped in this instance by the phenomena linked to fluctuating ambient temperatures.

## 4. Nanoparticles: Specificities

### 4.1. Sizes of Particulate Matter

In summary, and in connection with their name, the particles assimilated to dust can be categorized into three major groups, depending on their origin and chemical composition [52]:

- Coarse particles. These are defined as particles with a diameter of between 10 and 2.5 µm. This group is mainly associated with physical processes and mainly comprises particles that are formed following soil erosion, from pollen, or even from sea spray;
- Fine particles. These are defined as particles with a diameter of between 2.5 and 0.1 µm. These particles come directly from combustion processes, but they can also be formed in the atmosphere;
- Ultrafine particles. These are defined as particles with a diameter of less than 0.1 µm (PM 0.1). This group consists of particles resulting from combustion processes or that are formed from precursor gases.

**Figure 3:**
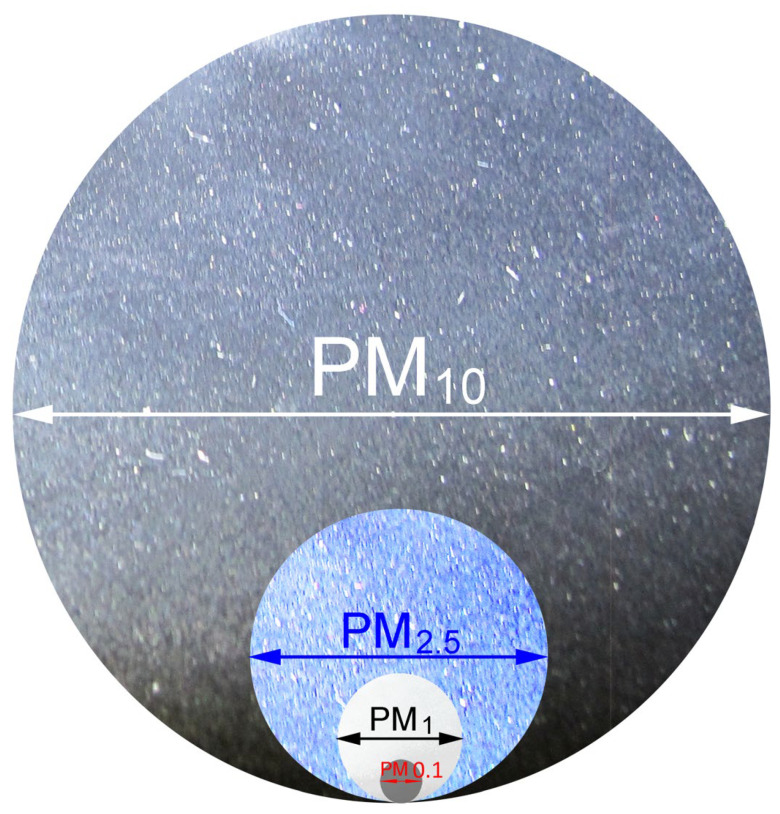
The range of particle sizes.

The smallest range of particles, ultrafine particles, consists of particles that are more than 100 times finer than PM 10 (for particles of the same chemical composition, toxicity increases with decreasing size) (ibid. [51,52]), as shown in Figure 3.

### 4.2. Emission Modes

**Figure 4:**
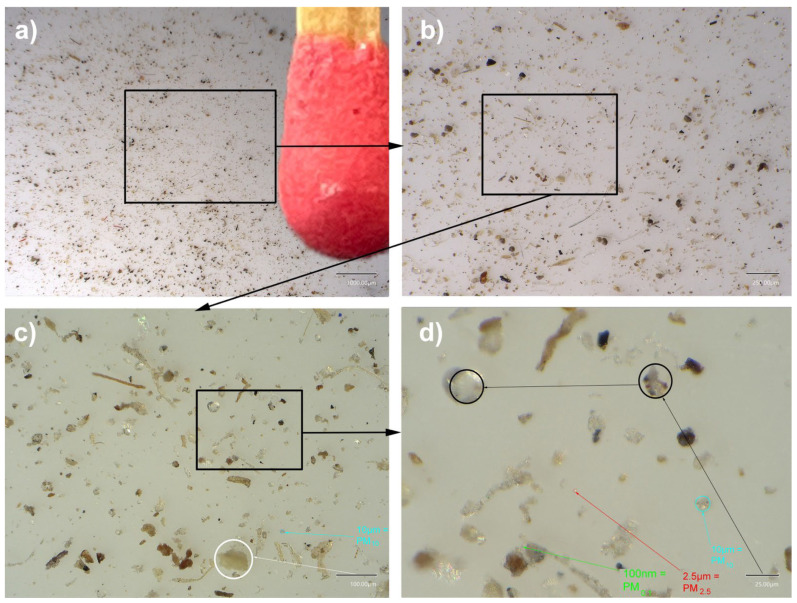
(**a**) Comparison scale under an ultra-high-accuracy 4 K microscope (Keyence VHX-7000); a matchstick head is shown for scale. Magnification 30×. (**b**) Detail of (**a**) at a magnification of 100×. (**c**) Detail of (**b**) at a magnification of 300×, where PM 10 (in blue) start to be identifiable. (**d**) Detail of image (**c**) at a magnification of 1000×: PM 2.5 starts to become visible (in red) but nanoparticles (PM 0.1 in green) are still hard to identify.

The emission modes and processes of UFPs and fine and coarse PM, as in the case of particles found in the atmosphere (ibid. [52,53,54]), can be classified according to the size of the particles that are in the majority, compared with the others: they are identified by the procedure shown in Figure 4.

There are three main identification modes (ibid. [51]):

- **Nucleation mode** (the mechanism by which a gas is converted into particles under specific conditions, resulting in the formation of secondary particles): this mode corresponds to ultrafine particles with a diameter of less than 100 nm. Particles of this size are mostly secondary and are formed by the process of nucleation, hence their name.
- **Accumulation mode**: this mode corresponds to fine particles with a diameter of between 100 nm and 2.5 μm. Particles of this size can either be directly emitted or are from finer particle-magnification processes. Their name comes from their long lifespan in the atmosphere, which allows their accumulation.
- **Coarse mode**: this mode corresponds to coarse particles with a diameter of between 2.5 and 10 μm. These particles are primarily particles emitted directly by natural and anthropogenic sources (such as abrasion processes and sea salt) (ibid. [52])

Although ultrafine particles constitute a small mass of PM (from 1 to 8%), they are the most numerous in a given volume and have a larger reactive surface than coarse particles. It takes one million UFPs (PM < 100 nm) to reach the mass of a 10 μm particle (PM 10) [55].

Ultrafine particles/nanoparticles account for 80% of the concentration in the number of particles in an urban environment [56,57], which probably partly explains their cumulative increase in conservation problems linked to soil-trapping, as highlighted in this study.

### 4.3. Particulate Matter Chemistry

In terms of their chemistry, fresh particles, depending on their production mode, are composed of sulfuric acid, soot, organic matter, or trace elements. UFPs also contain PAHs (polycyclic aromatic hydrocarbons), which mainly come from the pyrolysis–pyrosynthesis of organic matter (fossil fuels, wood, etc.), as well as from unburnt matter. Two types of PAH can be distinguished: petrogenic, which refers to the hydrocarbons present in crude oil, i.e., of natural origin, which are characterized by a high proportion of branched hydrocarbons; and pyrogenic, which refers to those hydrocarbons produced by the combustion of organic matter that is rich in carbon, i.e., fossil fuels or wood (ibid. [57]). As well as PAHs, UFPs contain VOCs, SO_2_, and NOx.

Pollution derives from outdoor or indoor sources (note that indoors, pollution coming from the outside adds to the pollution generated indoors): organic carbon, metallic trace elements (ETM), sulfates, EC (elemental carbon), and sometimes ammonium and nitrate. Ultra-fine particles can have unique physicochemical properties that are relevant to health and disease; coal-derived UPFs are rich in Na, K, Mg, Ca, Ti, Mn, Fe, Co, Ni, Zn, V, Cr, Cu, Sb, As, Se, S, and Cl (ibid. [45,58,59,60,61,62,63,64]).

The accumulation mode is not composed solely of UFPs, unlike in the nucleation mode [65]. According to their different formation processes, the compositions of the two modes are different.

Finally, it should be noted that soot nanoparticles result from incomplete combustion, involving numerous physico-chemical phenomena. From the initial fuel to the final fractal aggregate, the various successive stages of pyrolysis, nucleation, surface growth, coalescence, aggregation, and oxidation produce soot particles, which result from incomplete combustion. Depending on the combustion conditions, this can produce various proportions of organic compounds (OC/TC = organic carbon (OC) over total carbon (TC)). Moreover, when these nanoparticles are emitted into the atmosphere, volatile organic compounds can be adsorbed, forming a gangue around these fractal aggregates. However, the exact mechanism involved in the passage of PAHs to the first soot nuclei is still not completely known (ibid. [52]).

## 5. Forces Acting on Coarse Dust of PM 10 and PM 1.5

### 5.1. Nature of the Forces at Play

The “dustiness” of a powder, i.e., its potential to generate dust, can be considered a property of the powder, which emits dust when it is handled or put under stress. Granular materials have the particular quality of being able to behave like a solid, a liquid, or even a gas, depending on the stress imposed (Jaeger and Nagel, 1996) (ibid. [60]). The forces that are exerted on dust all depend on its radius, either directly or by the intermediary of the electric charge. Thus, these forces can be classified into two categories: those independent of the load and those determined by it.

In the category of load-independent forces (ibid. [59]) are:

- The force of gravity;
- The force of friction or drag force (in the case of gas flow) of the neutrals;
- The force of thermophoresis, driving the dust toward colder zones.

In the category of load-dependent forces are:

- The electric force;
- The ionic driving force [66], (ibid. [41])
- The force of interaction between particles of dust.

Thus, whether via the charge or directly via the radius, the size of dust particles is decisive for their dynamic behavior (ibid. [66]).

### 5.2. Deposition of the Particles

**Figure 5:**
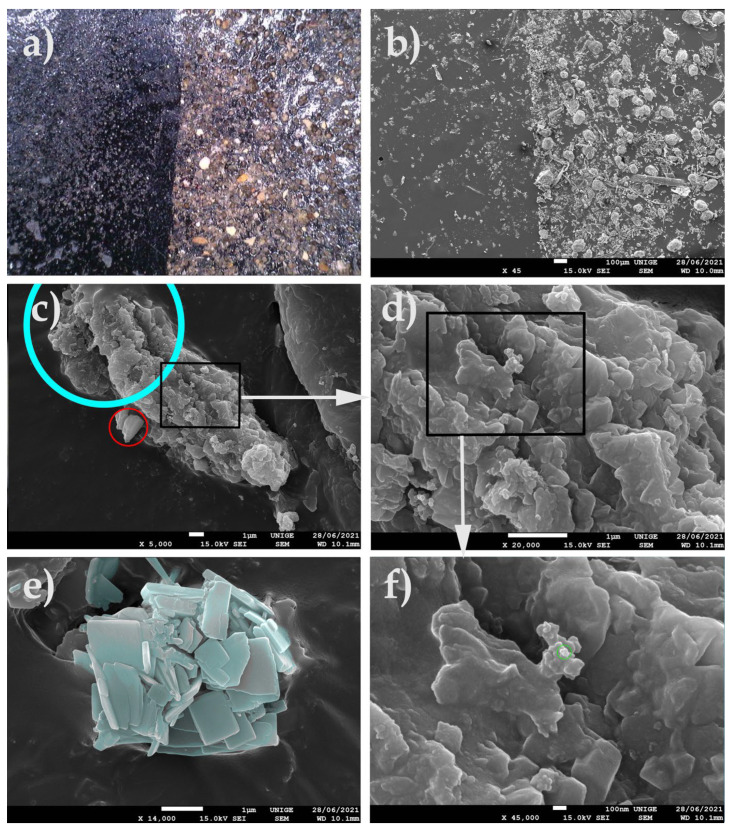
Examples of various sizes of particles and the nature of dust collected on the back of an artwork. (**a**) Dust deposited on an SEM stub (carbon tapes): coarse particles on the right part, thin particles on the left. Magnification average 40×. EDS (energy-dispersive X-ray spectroscopy) analyses revealed various compositions: C, N, O, Na, Mg, Al, Si, S, K, Ca, and Fe. See Table 1. (**b**) View of particles at an SEM magnification of 45×, scale 100 µm. (**c**) Scale 1 µm: PM 10 (in blue) and PM 2.5 (in red) can be identified. Magnification 5000×. (**d**) Particles at a magnification of 20,000×. (**e**) Aggregates of salt crystals, identified in turquoise, at a magnification of 14,000×. EDS analyses have revealed various compositions: C, O, Si, S, K, and Ca. (see (**e**,**f**)) At a scale of 100 nm, magnification 45,000×, examples of PM 0.1 nanoparticles are identified in green.

Fine dust is not a homogeneous substance but instead consists of a variety of primary and secondary particles. The particles vary in size, shape, and density, as well as showing very varied states of aggregation and chemical compositions (see Table 1 and Figure 5). Often, the finest particles quickly agglomerate with larger particles or land on various surfaces. Coarse particles, in contrast, are deposited relatively quickly by sedimentation or by impaction (fracturing into several fragments that fit together into each other). It is the median fraction, from 0.1 to 1 μm, which remains in suspension for the longest period (up to a week) and can therefore be transported over long distances [67].

Notably, higher accumulation is caused by fine particles on vertically oriented indoor surfaces due to Brownian motion and diffusion, whereas the large amounts of dust formed by coarse particles deposited on horizontal surfaces are attributed to gravitational sedimentation; in both cases, the phenomenon is relatively rapid. In addition, the size of the particles and the chemical components together play a role in the deposition mechanisms (ibid. [29,41,67]).

Electrostatic forces must also be considered because the surfaces of nanoparticles carrying charges exert interactions among each other: repulsion occurs when the charges are identical, which, in the case of particles, is equivalent to maintaining their autonomy and preventing their aggregation or agglomeration; attraction occurs when the charges are opposite, which accelerates the phenomenon of coagulation. These electrostatic forces are stronger in air than in water. Coagulation is only possible when the concentration of particles is sufficient, usually above 10^5^ p.cm^3^ (ibid. [64]).

## 6. Propagation in the Environment of PM 0.1 Nanoparticles

### 6.1. Propagation

The release of ultrafine particles into the environment occurs in the form of aerosols or hydrosols at the time of their production; they also form due to aerosols at the time of the handling of powder contents (ibid. [33]); they occur from abrasion or aging of the structures that carry them, for example, in tire wear; by release, for example, from liquid or viscous preparations applied to the skin; or by the discharge of liquid or gaseous effluents by production or treatment facilities (carried in packaging, filters, etc.) (ibid. [47]).

### 6.2. Diffusion

Ultrafine particles and gaseous precursors emitted into the atmosphere exhibit a particular behavior that is governed by seven major mechanisms (ibid. [51,68,69,70,71,72,73,74,75,76,77,78,79,80,81,82):

- Dilution (the mixing of emitted ultrafine particles in a larger volume of air);
- Nucleation (an important source in the formation of ultrafine particles from precursor gases). The conversion of gas into particles is the origin of secondary ultrafine particles (the source of UFPs);
- Condensation (gases can condense to form new particles, which are sources of UFPs);
- Coagulation (this mechanism causes the UFPs to grow with time and distance);
- Evaporation (the opposite mechanism to nucleation);
- Dry deposition (the mechanism in the nucleation mode is mainly related to sedimentation, which has no effect on the finest particles);
- Wet deposition (related to the deposition of particles from the atmosphere by precipitation: rain, snow, or hail).

These ultrafine particles agglomerate to form variably sized clusters, but the diameter does not generally exceed 1 µm (ibid. [76,77,78,79,80,81,82]).

## 7. Nanoparticles and Specific Surfaces

### 7.1. Specific Surface Area

**Figure 6:**
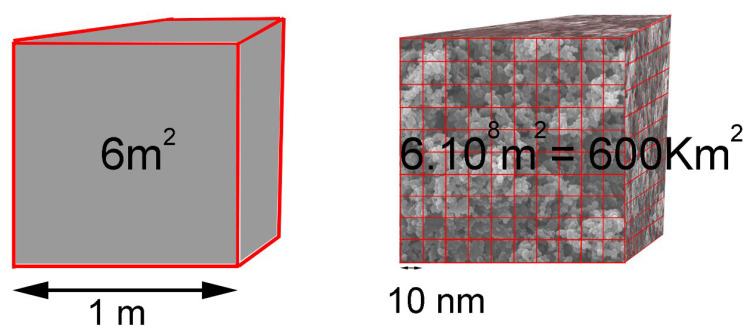
The same volume (6 m^2^), when constituted of nanocubes of 10 nm per side, develops a surface area of 600 km^2^ (according to Sophie Carenco in *Chimie et Nano/Une question d’échelle!* (CultureSciences-Chimie)) (ibid. [76]).

The number of atoms constituting a nanoparticle is small; therefore, the proportion of atoms exposed on its surface is relatively large, compared with that of a macroscopic material (ibid. [76,77,78,79,80,81,82,83,84,85]. As a result, one gram of nanoparticles develops an incomparably larger surface area than one gram of common particles, such as grains of sand. As an example, let us take a cube with sides of one meter. This cube has a surface of 6 m^2^ in a single block. For the same volume of 6 m^2^, but when that cube is made up of nanocubes with 10 nm edges, the surface area would be 600 km^2^ (ibid. [76]) (Figure 6), which is equivalent to the surface area of the city of Mumbai. Another example is that activated carbons may develop very high specific surface areas (more than 1000 m^2^/g), whereas non-porous glass balls will not develop specific surfaces that are greater than 1 m^2^/g (ibid. [85]); we can also add that a spherical nanoparticle with a diameter in the order of 2 nm, for example, has 50% of its constituent atoms on the surface (ibid. [76]). To use the example quoted in the Publifocus information brochure (Cerutti, 2006), “a cube with an edge length of 1000 nm is made up of approximately 1 billion molecules, of which 0.6% are on the surface. A cube with a side length of 10 nm only has 1000 left, but 50% of them are on its faces”. As shown in Figure 6, this modification of the surface/volume ratio greatly increases the reactivity of the particles (ibid. [76]).

### 7.2. Chemical Reactivity

As has been demonstrated, any phenomenon linked to the surface of a material is, therefore, exacerbated in the case of nanoparticles (ibid. [82]). The reactivity, in particular the chemical reactivity, of a particle depends on its surface area: the surface/volume ratio increases by a factor of 10 when the dimensions are divided by 10; the proportion of atoms or molecules on the surface, compared with the total number of atoms or molecules, increases in the same ratio (ibid. [38]). Due to their large specific surface area, the adsorption of potentially toxic compounds on the surface is favored (for example, a metal that is reluctant to form chemical bonds will gain in reactivity when it is reduced to the nanoscale (ibid. [35])). Certain compounds, such as metals or polycyclic aromatic hydrocarbons (PAHs), can therefore be adsorbed on the surface of the particles (ibid. [35]). The interaction forces increase as the size decreases, but knowledge of the forces of adhesion must, however, be further elucidated (ibid. [35]).

### 7.3. Trapping of VOCS and PAHs

The cocktail effect should, therefore, not be neglected because these VOCs and PAHs are grafted onto nanoparticles that have an ability to adorn themselves with a crown of complex molecules, drawn upon during their emission: this is called the “corona effect” (the longer the molecules stay in the environment, the more they charge their crown of molecules) (ibid. [31]). PAHs are semi-volatile compounds, i.e., they are capable of changing from the gaseous state to the particulate state; they are organic compounds that are formed during the pyrolysis reactions of a hydrocarbon.

These PAHs are therefore precursors of soot, which can still exhibit increases in primary particles (depending on the nature of the compounds resulting from incomplete combustion) along with a non-negligible quantity of organic compounds in volume (by absorption) or on the surface (by adsorption) (ibid. [79]).

## 8. Case Studies

### 8.1. Packaging and Materials That Act as Dust Magnets: Triboelectric Effect

#### 8.1.1. Attraction

**Figure 7:**
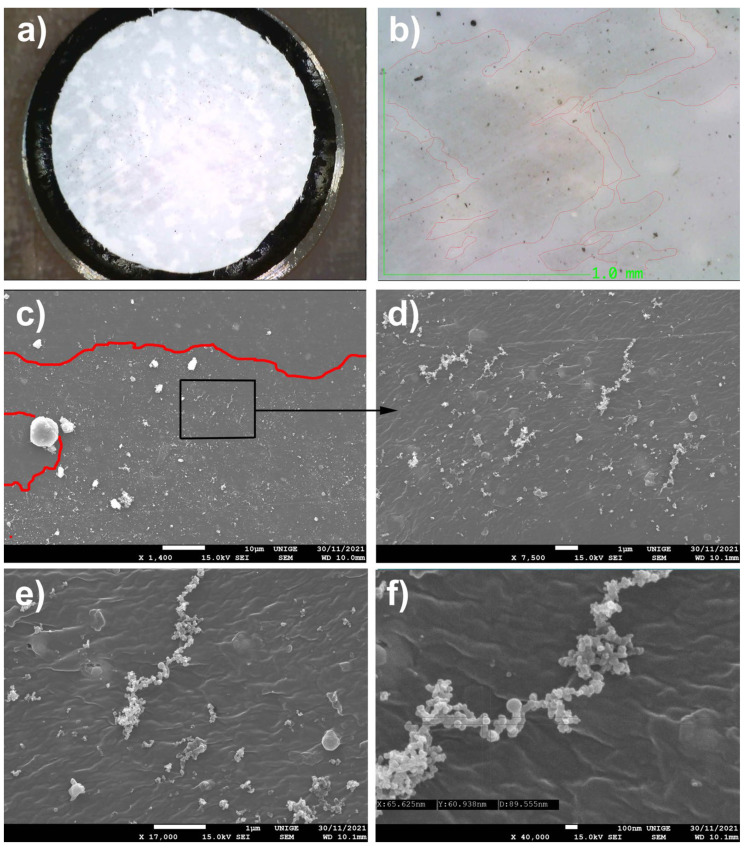
(**a**) Sample of a polyethylene sheet (LDPE) on a stub, with aggregates of thin dust (magnification 20×). (**b**) Localization of thin particles as a greyish halo (located in red: magnification 220×). (**c**) SEM pictures at a magnification of 1400×, scale 10 µm. (**d**) SEM pictures at 1 µm scale, magnification 7500×. (**e**) SEM pictures at a magnification of 1700× show clearly branched aggregates of nanoparticles, in the shape of spheres, present on this LDPE sheet. (**f**) Nanoparticles, measured with an SEM and at a magnification of 40,000× at the 100 nm scale: the average diameter is around 89 nm.

The very common observation of dust maintained on the surface of plastic films of low-density polyethylene (LDPE) or of bubble wrap, as is often used in the packaging of works of art, led us to undertake SEM analyses of pieces of film from polyethylene packaging, in order to visualize the extent and nature of the deposits of PM at the microscopic and nanometric level (Figure 7).

#### 8.1.2. Artwork Packing Material

We observed that clusters of blackish dust and greyish deposits of nanometric appearance on a sample of low-density PE packaging film seemed to have been deposited by convection and by triboelectricity induced by the nature of the substrate [83,84,85,86,87,88,89,90]. These types of PM 10, PM 2.5, and PM 0.1 deposits are characteristic of the aggregates of fine and very fine dust that accumulate on packaging that is naturally charged. On the surface, these phenomena of the nucleation of ambient nanoparticles and the growth of aggregates in situ have been widely observed: the primary particles assembled either due to weak van der Waals bonds (agglomerates, or primary particles bonded together by weak forces) or due to strong covalent bonds (aggregates, or primary particles strongly bound together) (ibid. [83]). Depending on the conditions of the medium (pH, temperature in °C, etc.), nanoparticles can, therefore, be found either in the form of free primary particles or in the form of agglomerated or aggregated primary particles (ibid. [83]).

### 8.2. Materials Acting as Dust Electromagnets, Using Thermal Convection and Triboelectricity

#### Thermal Convection and Dust

In order to quantify the impact of ambient sources of dust, nano-like dust from an old neon tube reflector sourced from an art warehouse has been collected.

**Figure 8:**
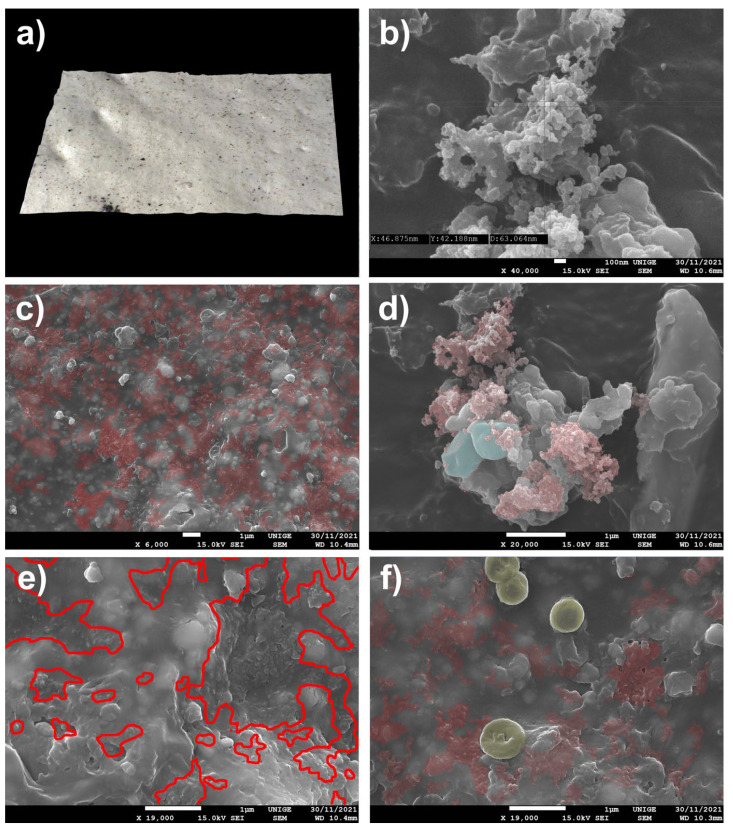
(**a**) A sample taken from an acrylic mock-up, soiled by the collected dust and then deposited on a surface, observed under an ultra-high-accuracy 4 K microscope. Magnification scale, 100×. (**b**) Nanoparticles, measured with an SEM under 40,000× magnification at 100 nm scale: the average diameter is around 63 nm. (**c**) Extensions of PM caught in the acrylic surface, identified in red in the SEM picture, at a magnification of 6000×. (**d**) Agglomerates of the nanoparticles located are identified in red in the picture: the salt micro-crystals contained in these dust samples are depicted in blue. Magnification scale, 20,000×. (**e**) Extension of nanoparticles caught in the acrylic surface (located in red in the SEM picture). Magnification scale, 19,000×. (**f**) Trapping areas of nanoparticles and the presence of molds, identified in yellow in the SEM picture, at a magnification of 19,000×.

The apparent fineness of the deposit seemed to be characteristic of the small size of the particles produced therein by thermal convection in the air, which mixed the dust [91]; Figure 8).

### 8.3. Mock-Up

Samples were applied to a mock-up of a white acrylic paint layer (titanium white n° 191, Lascaux Artist©, Brüttisellem, Switzerland) placed in moderate-temperature conditions for a few weeks.

#### Observations

Examination of the deposits via SEM enabled us to attest to their mainly nanometric nature, and also to notice the presence of micro-crystals of salts and molds, agglomerated with nanometric and micrometric dust.

Secondly, the appearance and arrangement of the nano-PMs trapped in the acrylic surface have been noted: they are indicative of indelible dirt that is partially trapped in the thickness of the pictorial layer of the mock-up.

In the Figure 8b, the nanometric-sized dust (see measurements) is anchored halfway down the body, caught in the obvious stickiness inside the pictorial material itself.

This provision implies the obvious impossibility of appropriately cleaning a pictorial layer that has been impacted in this way.

### 8.4. Case Study of Trailing Wires and Spider Webs with Adhered Nano Dust

#### 8.4.1. Spiders

Trailing threads or cobwebs can carry or become loaded with dust, among other nanoparticles [92].

**Figure 9:**
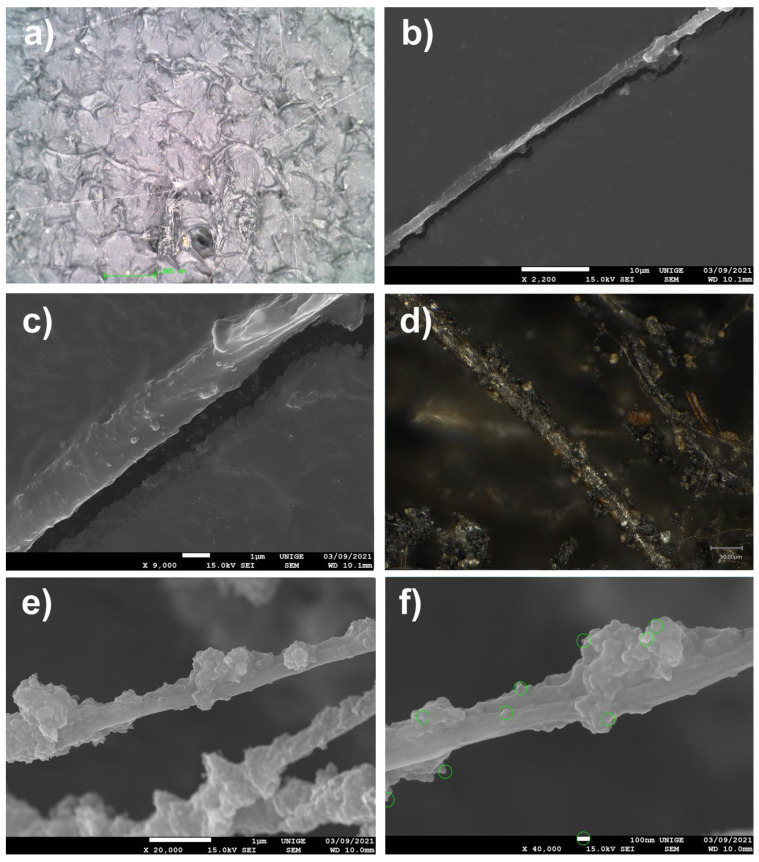
(**a**) Trailing wires adhered to a monochromatic black painted surface, at a magnification of 55×. (**b**) Spider wires, observed with SEM, at a magnification of 2200×. (**c**) At a magnification of 9000×, accumulations of particulate matter adhered to the thread start to be visible. (**d**) Microscope viewing (ultra-high-accuracy 4 K microscope) of a spider thread coming from a ventilation exit inside a car: it seems to carry many nanoparticles that are soiled by HAPs. Magnification, 500×. (**e**) At a magnification of 20,000×, the SEM clearly reveals aggregates of nanoparticles caught on the thread. (**f**) Measurements (in green) of some nanoparticles under SEM, at a magnification of 40,000× at a 100 nm scale.

The observation of submicronic particles stuck by simply becoming trapped in the ambient air on the threads of cobwebs makes it possible to identify not only the microparticles but also the nature of nanoparticles, which are clearly visible at a scale of 100 nm (Figure 9).

#### 8.4.2. Mock-Up

Samples were directly applied onto carbon tapes of SEM specimen stubs, in order to obtain a good distribution and enough variety in the dust-grain specimens.

#### 8.4.3. Observations

Most of the particles measured were obviously nanometric in size, with an average size of around 50–60 nm.

These natural ambient dust traps capture particles floating in ambient air and can also contribute to the surface soiling of pictorial layers, first by direct deposition, then by the migration of nanoparticles by simple contact.

### 8.5. Lucas Samaras Dusty Sculptures

#### 8.5.1. Sculptures and Dust

**Figure 10:**
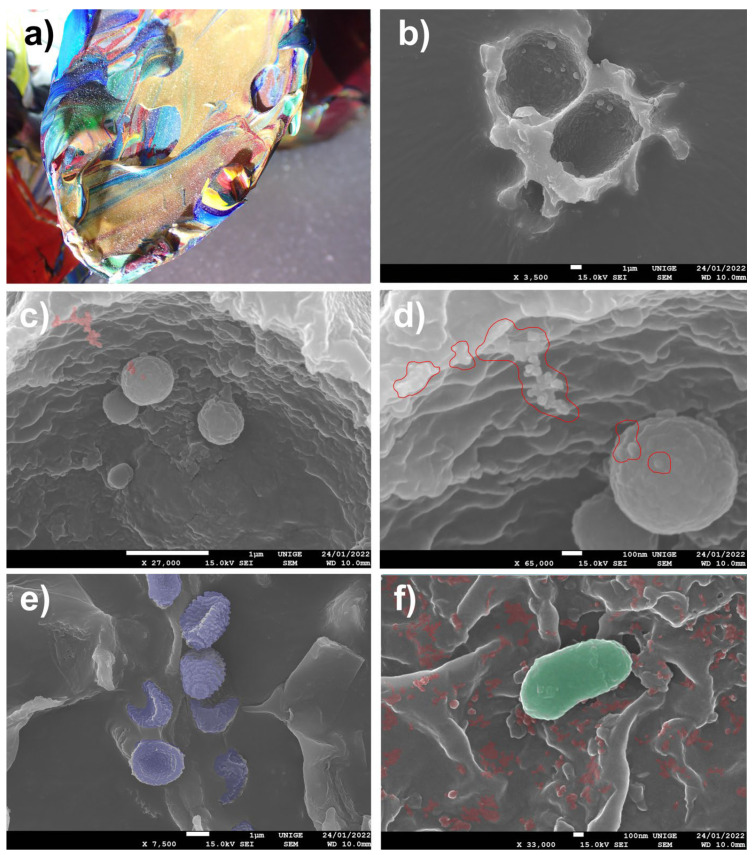
(**a**) Preview of a dusty area on the sculpture with acrylic impasto. (**b**) A large, hollow particle of dust (20 µm) adhered to the carbon stub, with smaller particles inside, at a magnification of 3500×. (**c**) At an SEM magnification of 2700×, branched aggregates of nanoparticles of a spherical shape can be identified: they are caught on the sides of the biggest particle (identified in red). (**d**) Aggregates of nano-PMs, surrounded in red in the SEM picture, at a magnification of 65,000× (surrounded in red). (**e**) Examples of pollen particles (surrounded in purple), taken from the dust sample observed through SEM, at a magnification of 7500×. (**f**) Identification of bacteria, surrounded in green in the SEM picture. Nanoparticles caught on the larger PM, surrounded in red in the SEM picture, at a magnification of 33,000× at a 100 nm scale.

Two works by Lucas Samaras have been entrusted to us, in order to clean away the heavy surface dust: the deposits are caught in the entanglements of the acrylic impasto that the artist has applied to the manufactured elements (knives, a spoon, and a metal fork) that make up the sculptures (Figure 10).

#### 8.5.2. Mock-Up

The relatively thin nature of the dust deposited on the surface meant that analyses were necessary (because it was not possible to collect a sample directly from the artwork).

#### 8.5.3. Observations

The surface dust collected was dusted directly onto the carbon tapes of SEM specimen stubs, in order to obtain a good and sufficiently diversified distribution of the dust-grain specimens.

A mixture of mineral particulate matter, pollens, mold, and bacteria of about one micron can be observed.

The nature of the carbon adhesive has absorbed the finer dust particles by embedding them in the adhesive mass; fortunately, islands of coarse PM harbored finer particles and a certain number of symptomatic nanoparticles.

### 8.6. Experimentation of Acrylic Pictorial Layer Mock-Up Specimens

#### 8.6.1. Mock-Up: Nature

In order to more precisely discern the processes of the surface penetration of nanoparticle dust, a series of mock-ups of an acrylic paint layer (titanium white n° 191, Lascaux Artist©, Brüttisellem, Switzerland) were carried out, with paint applied to a thickness of 500 μm, produced using an applicator film shot on a cardboard canvas intended for artists and then left to dry for several months.

**Figure 11:**
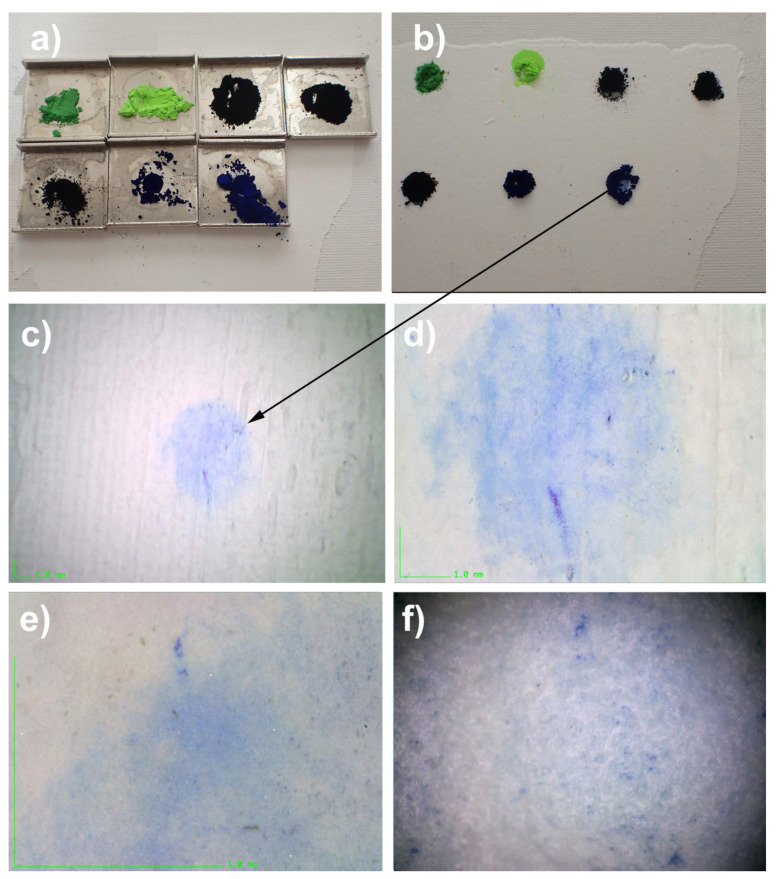
(**a**) Calibrated deposition of pigmentary powder on an acrylic mock-up that is 500 μm thick. (**b**) Powders were left in place for 60 days. (**c**) After removing and cleaning, spontaneous penetration traces of the pigment PB15 in this area are very clear. Magnification 20×. (**d**) Magnification 55×. (**e**) Magnification 220×. (**f**) Magnification 740×.

A choice of pigments among a series of fine particle-sized pigments and fingerprinting powders (as used in forensics) was simply deposited on the surface of the mock-up and left in place for two months in a normal workshop atmosphere (Figure 11).

#### 8.6.2. Powder Choice

**Figure 12:**
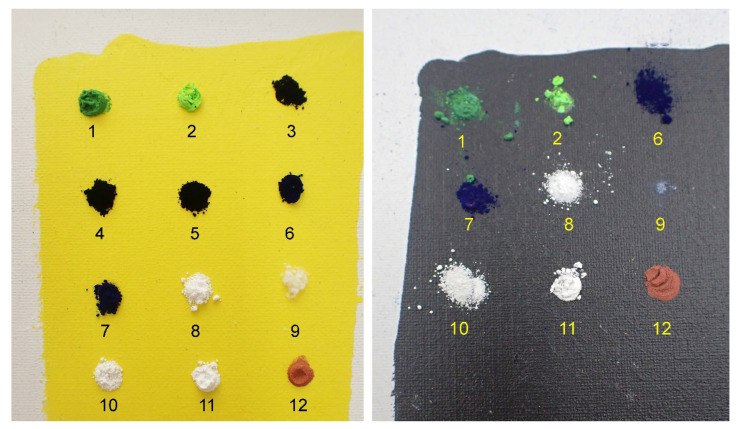
(1) Lightning powder: Latent Print Powder Pak—Greenwop. (2) Lightning powder: Blitz Green #1-0063. (3) Lightning powder: Black #1-0001. (4) Gas Channel Black (DSP Schwartz 4 Degusa) PBk7. (5) Lamp black: PBk6. (6) Prussian blue: PB27. (7) Phthalocyanine blue: PB15. (8) Zinc white: PW4. (9) Aerosil 200. (10) Rutile: titanium white PW6. (11) Lead white: PW1. (12) Fine copper powder at a concentration of 99.997% copper.

The pigments were chosen for their fine, or even very fine, grain size, approaching the size of the submicron particles in question in this study, including phthalocyanine blue and Prussian blue, which are classified in the range of 0.1 μm and as a gas in the 0.05 μm range. Fingerprint powders can be of different compositions: either magnetic and colored or black and combined with a number of compounds, such as lycopodium, graphite, charcoal, carbon black, and photocopier toners. Toners are made up of carbon black nanoparticles with a size that varies between 15 and 300 nm; they form agglomerates of a few tens of micrometers (ibid. [15]) (Figure 12).

#### 8.6.3. Protocol

After a deposition period of two months (without mechanical action or pressure of any kind), the pigments were aspirated; then, the area was treated with light and gentle exfoliation in an attempt to eliminate the pigment powders on the surface to the maximum level of cleaning possible.

In order to eliminate the traces of penetration (infiltration) of the still very visible powders, a trial (albeit unsuccessful) cleaning was conducted with a buffered aqueous solution of citrate (tri-natriumcitrat-2-hydrat, CAS 6132-04-3, Kremer Pigments, Aichstetten/Allgaü, Deutschland) with a pH of 7.00 and conductivity of 8000 µS/cm, with a DTPA (diethylenetriaminepentaacetic acid, CAS 67-43-6, Sigma-Aldrich Merck, Buchs, Switzerland) solution at pH 7 and conductivity of 6750 µS/cm, and with an EDTA (ethylenediaminetetraacetic acid, CAS 60-00-4, Sigma-Aldrich Merck, Buchs, Switzerland) solution at a pH of 8.5 with a conductivity of 4800 µS/cm.

#### 8.6.4. Observations

The visible penetration of most of the dusting powders on the mock-ups of pictorial layers made it possible to easily create samples in order to observe them under a digital microscope and, subsequently, via SEM (Laboratory University of Geneva Department of Earth Sciences, Geneva, Switzerland)

**Figure 13:**
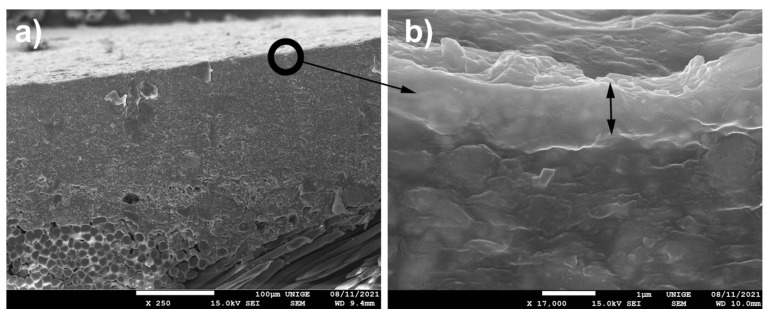
(**a**) Cross-section under SEM: we can see the more compact nature of the drying fronts, perpendicular to the surface, at a magnification of 250×. (**b**) Preview under SEM of the drying fronts, average 1 µm thick, at a magnification of 17,000×.

All the specimens had the deposited powders clearly trapped on the surface: the most telling specimens for carrying out the SEM examinations are shown in Figure 13.

#### 8.6.5. SEM

Scanning electron microscopy (SEM) sometimes required us to use the BEI (backscattered electron imaging) mode in order to analyze certain penetration areas that are more difficult to determine, because the color (black and white) on the micrograph obtained is a reconstruction obtained via an electronic system and has nothing to do with the colors of the object examined [93]. Indeed, it was more difficult than we expected to identify the nanoparticles trapped in the pictorial layer, although they were very visible to the naked eye; on these pictorial layers, the surfaces looked so smooth and yet appeared so textured under electron microscopy.

The perpendicular sampling section of the surface also made it possible to visualize the slightly more compact nature, (about 1 µm from the drying front), which seems to be more concentrated in acrylic polymers (in addition to the probable exudations of surfactants on the surface) and less loaded with grains (fillers and pigments). This drying front, as a “wall” limits the “freedom” of channels due to broadcast restrictions [94,95,96]. This effect tells us that in addition to the lateral drying front, an accumulation of particles occurs on the surface of the film during drying (ibid. [94,95,96]).

It is possible that the viscoelastic characteristics and the influence of the glass transition temperature of the thermoplastic binder are therefore more pronounced (Figure 13).

#### 8.6.6. Optical Microscopy and Cross-Sections

**Figure 14:**
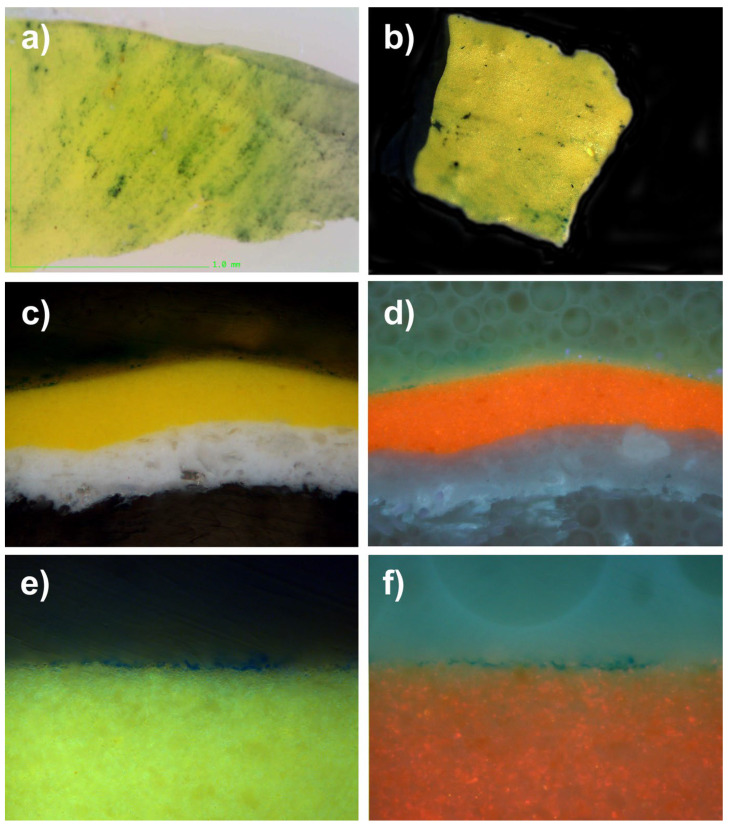
(**a**) Under a digital microscope at a magnification of 220×, this is the sample of an acrylic painted mock-up (cadmium yellow light PY35, n° 112, Lascaux Artist©, Brüttisellem, Switzerland) with a layer of phthalocyanine blue (PB15), after its removal. (**b**) Sample of this mock-up, derived by the laboratory for the cross-section. (**c**) Picture taken under optical microscopy of the cross-section of the micro-sample (lens: MPlan 20×/0.45): we can see the penetration of the pigment inside the layer of the acrylic mock-up, which prevents the complete cleaning of the surface; this looks like some sort of tattoo. (**d**) The same cross-section, observed under UV lighting, enables the identification of the penetration of the pigment. (**e**) Enlarged view of the areas penetrated by pigment PB15 (lens: MPLan 50×/0.85). (**f**) Enlarged view of the areas penetrated by pigment PB15 (lens: MPLan 50×/0.85), seen under UV lighting.

**Figure 15:**
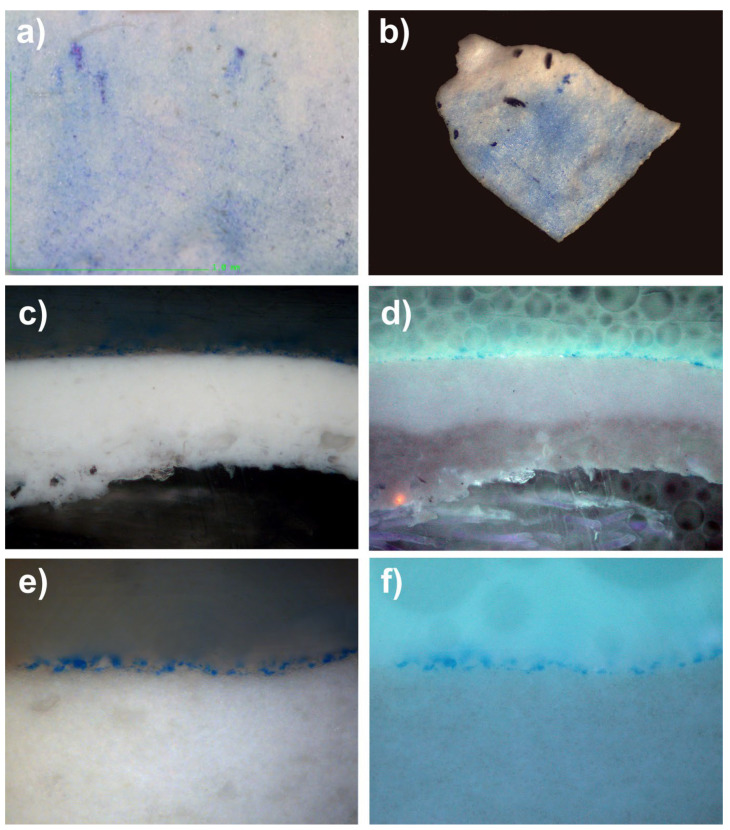
(**a**) Under digital microscopy, at a magnification of 220×, this is a sample of an acrylic painted mock-up (titanium white n° 191, Lascaux Artist©, Brüttisellem, Switzerland) with a layer of Prussian blue (PB27), after its removal. (**b**) Sample of this mock-up, performed by the laboratory for the cross-section. (**c**) Picture taken under optical microscopy of the cross-section of the micro-sample (lens: MPlan 20×/0.45): the penetration of the pigment is visible inside the layer of the acrylic mock-up, which prevents the complete cleaning of the surface; this looks like some sort of tattoo. (**d**) The same cross-section, observed under UV lighting, enables the identification of the penetration of the pigment. (**e**) Enlarged view of the areas penetrated by pigment PB27 (lens: MPLan 50×/0.85). (**f**) Enlarged view of the areas penetrated by pigment PB27 (lens: MPLan 50×/0.85), under UV lighting.

**Figure 16:**
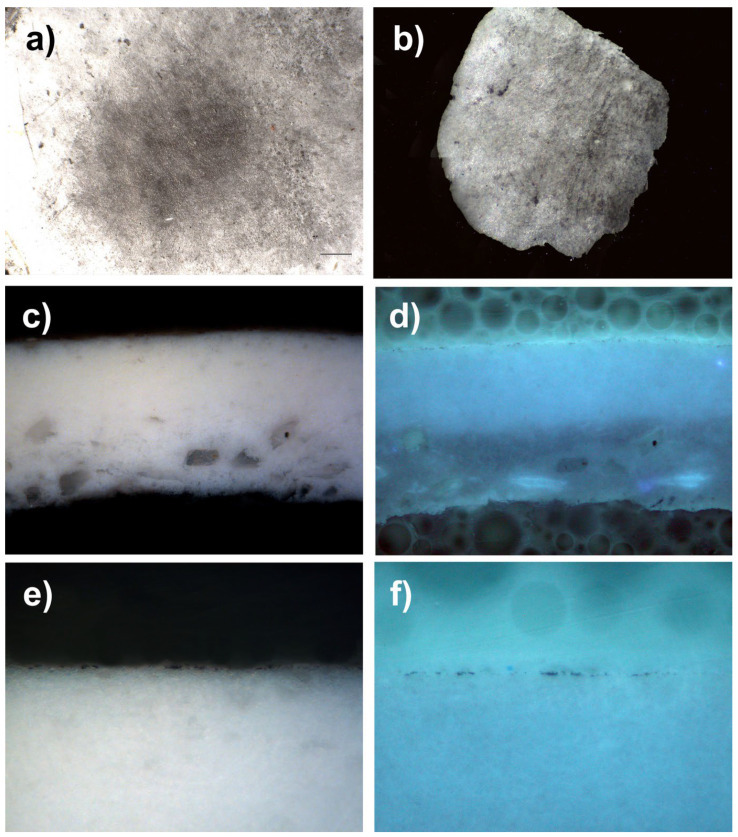
(**a**) Under a digital microscope, magnification 220×, is a sample of an acrylic painted mock-up (titanium white n° 191, Lascaux Artist©, Brüttisellem, Switzerland) with a layer of black fingerprint powder from lightning powder, after its removal. (**b**) Sample of this mock-up, performed by the laboratory for the cross-section. (**c**) Picture taken with optical microscopy of the cross-section of the micro-sample (lens: MPlan 20×/0.45): the penetration of the pigment is visible inside the layer of the acrylic mock-up, which prevents the complete cleaning of the surface; this looks like some sort of tattoo. (**d**) The same cross-section, observed under UV lighting, enables the identification of the penetration of the fingerprint powder. (**e**) Enlarged view of the areas penetrated by the fingerprint powder (lens: MPLan 50×/0.85). (**f**) Enlarged view of the areas penetrated by the fingerprint powder (lens: MPLan 50×/0.85), under UV lighting.

To be able to better appreciate the penetration of the particles within an acrylic-type pictorial layer, we asked a specialized laboratory (Arte-Lab SL, Madrid, Spain), to produce optical microscope images of the cross-sections of several micro-samples. In this way, we were able to perfectly observe the powders, which penetrated the epidermis of the acrylic mock-ups. When observed under UV light, the grains of pigments are more distinctly visible because their fluorescence makes it possible to clearly differentiate them from the white mass of the acrylic mock-up of titanium oxide (titanium white n° 191, Lascaux Artist©, Brüttisellem, Switzerland), or from that of the acrylic mock-up of cadmium yellow (cadmium yellow light PY35, n° 112, Lascaux Artist©, Brüttisellem, Switzerland), or even the black mass of the ivory black acrylic mock-up (carbon black PBk7, n° 181, Lascaux Artist©, Brüttisellem, Switzerland) (see Figure 14, Figure 15 and Figure 16).

### 8.7. Mock-Up PW6 and Black Gas Channel SP Schwarz 4 Degusa

#### 8.7.1. Mock-Up

**Figure 17:**
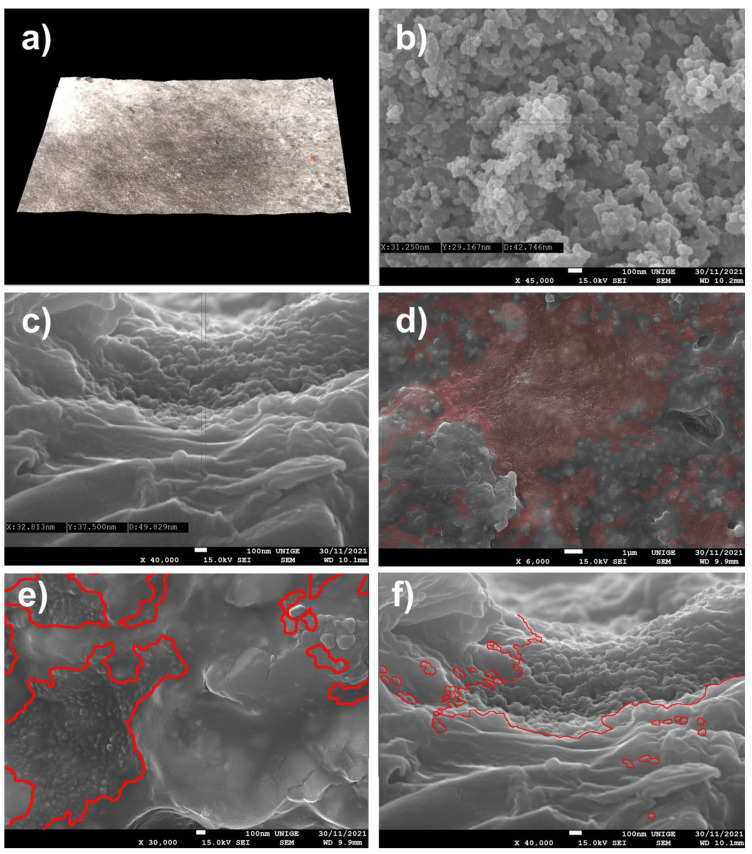
(**a**) View of a sample under an ultra-high-accuracy 4 K microscope: sample of an acrylic painted mock-up (titanium white n° 191, Lascaux Artist©, Brüttisellem, Switzerland) with a layer of carbon black (PBk7), after its removal. Magnification 100×. (**b**) Measuring of the pigment at SEM before its deposit, at a magnification of 40,000× at 100 nm scale: the average diameter of a particle is around 43 nm. (**c**) Measuring the pigment trapped in the mock-up at SEM, at a magnification of 40,000× at 100 nm scale: the average diameter of the particle is around 49 nm. (**d**) Agglomerates of nanoparticles trapped in the acrylic surface, identified in red in the SEM picture. Magnification 6000×. (**e**) Extension of the nanoparticles caught in the acrylic surface, surrounded in red in the SEM picture. Magnification 30,000×. (**f**) From a different area, a view of the nanoparticles of pigment trapped in the layer (surrounded in red). Magnification 40,000×.

The pigment was deposited on a mock-up of a white acrylic paint layer (titanium white n° 191, Lascaux Artist©, Brüttisellem, Switzerland) placed in moderate-temperature conditions for a few weeks (Figure 17).

#### 8.7.2. Observations

Observation of the SEM images made it possible to visualize the topography of the acrylic pictorial surface, where the original aspect is relatively homogeneous, according to the magnification scale, but where certain zones show the trapping of the nanoparticles of a gas black channel PBk7 SP Schwartz 4 Degusa, in the form of micro-grains partially stuck in the thermoplastic material.

The average grain diameter of this pigment (carbon black of the channel type, a gas black SP Schwartz 4 from Degusa) is 5 to 30 nm [97], a particularly fine-grained carbon black. (The last channel black process plant in the United States was closed in 1976 (ibid. [11])). Samples were obtained in the form of soot by the incomplete combustion of natural gas, producing channel black with an average particle size of 10–30 nm, giving carbon with very fine spherical particles that closely resemble micro-soot aerosols from diesel engines (ibid. [97]), characteristic of this study.

These nano-fouling particles spread on the surface at a mid-depth of the pictorial layer and were observed in the micro-hollows and clearly appeared in the shape of visible micro-granulation, demonstrating different densities from the surrounding molecules. This situation makes them inaccessible to simple dusting or mechanical scrubbing, but they are even less available for superficial cleaning in the aqueous phase.

Sometimes, the identification and localization with SEM were completed by comparing the powdered pigment representing this surface infestation and the particle size with the granulometry constituting the pictorial layer (resin, filler, and pigments) to differentiate them.

### 8.8. Deposition of Fingerprint Powders on the Surface of an Acrylic Mock-Up Using Black Fingerprint Powder (Black # 1-0001 Lightning Powder (as Used in Forensics))

#### 8.8.1. Mock-Up

**Figure 18:**
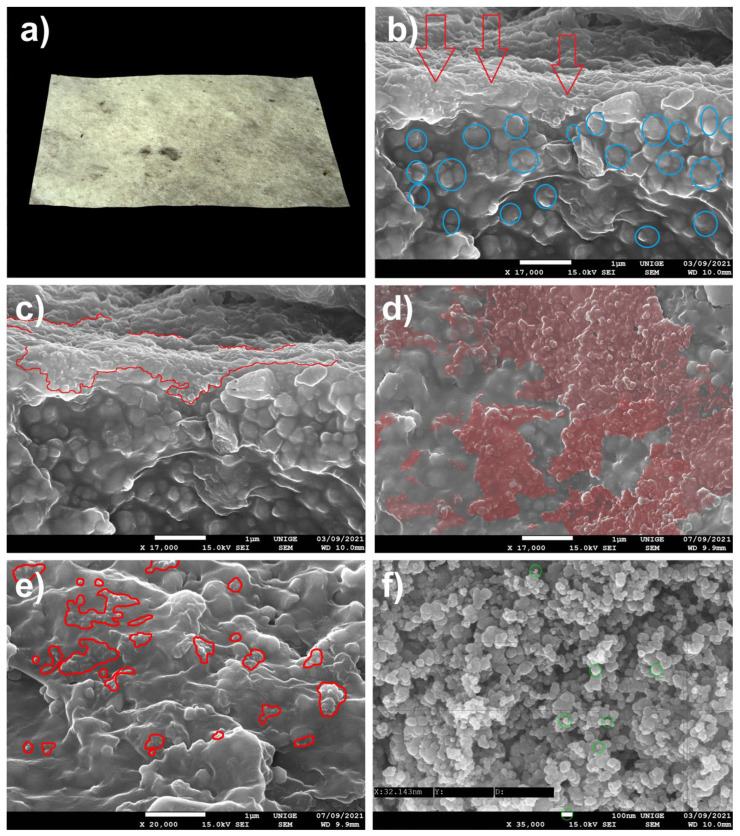
(**a**) View of a sample under an ultra-high-accuracy 4 K microscope: sample of an acrylic painted mock-up (titanium white n° 191, Lascaux Artist© Brüttisellem, Switzerland) with a layer of a black fingerprint powder, after its removal. Magnification, 200×. (**b**) Agglomerates of the nanoparticles of black fingerprint powder trapped in the acrylic surface, as indicated by red arrows in the SEM picture. We have identified some acrylic mock-up grains with blue circles that differ from the penetration of fine particles coming from the black fingerprint powder. Magnification, 17,000×. (**c**) Extension of the nanoparticles of black fingerprint powder trapped in the acrylic surface, surrounded in red in the SEM picture. Magnification, 17,000×. (**d**) Nanoparticle agglomerates trapped in the acrylic surface are identified in red in the SEM picture. Magnification, 17,000×. (**e**) Extension of nanoparticles trapped in the acrylic surface, surrounded in red in the SEM picture. Magnification, 20,000×. (**f**) Measurements with SEM of the particles of black fingerprint powder before its deposition, magnification 35,000×, at 100 nm scale: length, 32 nm; average diameter, less than 100 nm.

Fingerprint powders (ibid. [8]) were deposited on a mock-up of a white acrylic paint layer (titanium white n° 191, Lascaux Artist©, Brüttisellem, Switzerland) placed in moderate-temperature conditions for a few weeks, Figure 18.

In this case, the powder was applied by lightly rubbing the surface of the mock-up; then it was left without interference at room temperature for a few weeks. The deposit was more substantial and probably anchored more deeply using this method than by a simple surface application without mechanical action.

#### 8.8.2. Observations

The findings are very similar, in terms of the results obtained with the carbon black pigment (SP Schwartz 4 Degusa, gas black) at the level of the facies of the nanometric trapped on the acrylic pictorial layer of the mock-up.

The observation of the SEM images makes it possible to visualize the topography of the acrylic pictorial surface, where the original aspect is relatively homogeneous according to the magnification scale, but where certain zones exhibit trapping of the micro-grains, which are partially stuck in the thermoplastic material.

### 8.9. Micro and Nanopowder Dust of Silver Carbonate, Reduced with Formaldehyde on an Acrylic Mock-Up

#### 8.9.1. Mock-Up

**Figure 19:**
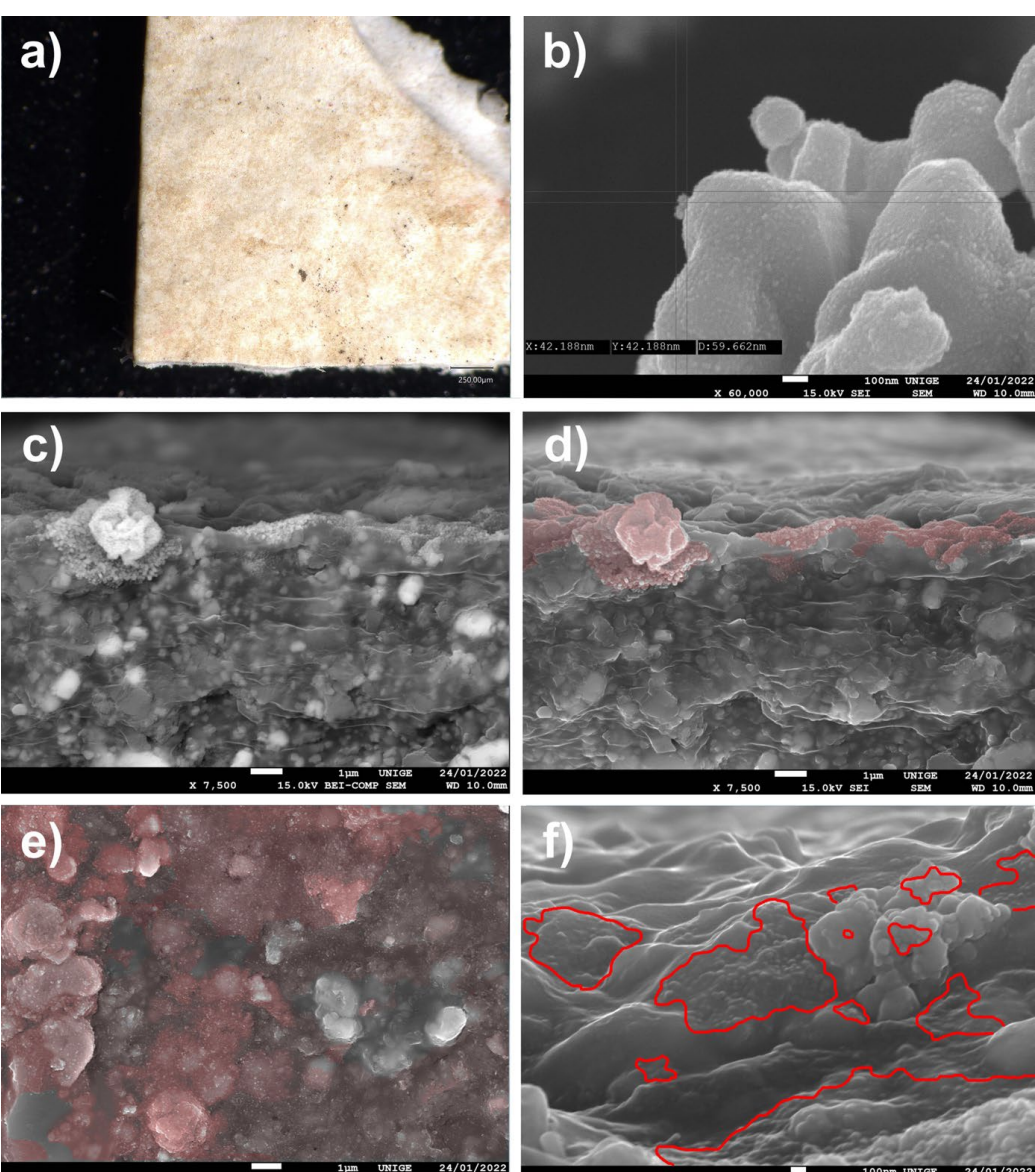
(**a**) View of a sample under an ultra-high-accuracy 4 K microscope: sample of an acrylic painted mock-up (titanium white n° 191, Lascaux Artist©, Brüttisellem, Switzerland) with a layer of powder dust of silver carbonate reduced with formaldehyde after its removal. Magnification, 100×. (**b**) Measuring of the powder dust of silver carbonate, reduced with formaldehyde, via SEM before its deposition, at a magnification of 35,000× at 100 nm scale: the average diameter of many particles was around 60 nm. (**c**) View with SEM of the powder dust of silver carbonate, reduced with formaldehyde, in BEI SEM mode, showing a different density of agglomerates of the caught particles. (**d**) The same area, seen in BEI mode, of the agglomerates of nanoparticles trapped in the acrylic surface, identified in red in the SEM picture. Magnification, 7500×. (**e**) Extension of nanoparticles trapped in the acrylic surface, identified in red in the SEM picture. Magnification, 7000×. (**f**) Nanoparticle agglomerates trapped in the acrylic surface, surrounded in red in the SEM picture. Magnification, 35,000×.

Silver carbonate, reduced with formaldehyde, was deposited on a mock-up of a white acrylic paint layer (titanium white n° 191, Lascaux Artist©, Brüttisellem, Switzerland) placed in moderate-temperature conditions for a few weeks (Figure 19).

The principal use of silver carbonate is for the production of silver powder, for use in microelectronics. metals. It is reduced with formaldehyde, producing silver that is free of alkali metals. The use of a partially nanometric Ag_2_CO_3_ silver carbonate powder, diameter 60 nm, with the BEI SEM made it possible to clearly distinguish a powder added on the surface of the acrylic mock-up: the difference in nature and density of the material could be clearly distinguished.

#### 8.9.2. Observations under BEI (Backscattered Electron Imaging) SEM Mode

The reading of the images in BEI mode indeed allowed for the easier location of the nanoparticle encrustations, as observed in the Figure 19c,d, using BEI1 and SEI2 with 7000× magnification, and BEI3 and SEI4 with 3700× magnification.

At the level of localization of the nanoparticles on the surface of the acrylic mock-up, we once again observed the same symptomatic partial penetration, along with irreparable staining of the pictorial layer, grouped in areas that are visible under a digital microscope or from normal observation of the surface.

### 8.10. Pictorial Layer of an Artwork by Kenneth Noland from 1973

#### 8.10.1. Sample from a Canvas Painted with Magna

Magna is the brand name of an acrylic paint, developed by Leonard Bocour and sold by Bocour Artist Colors, Inc., from 1947 until the mid-1970s. It was composed of pigments ground in an acrylic resin and dissolved in organic solvents, such as turpentine or mineral spirits.

**Figure 20:**
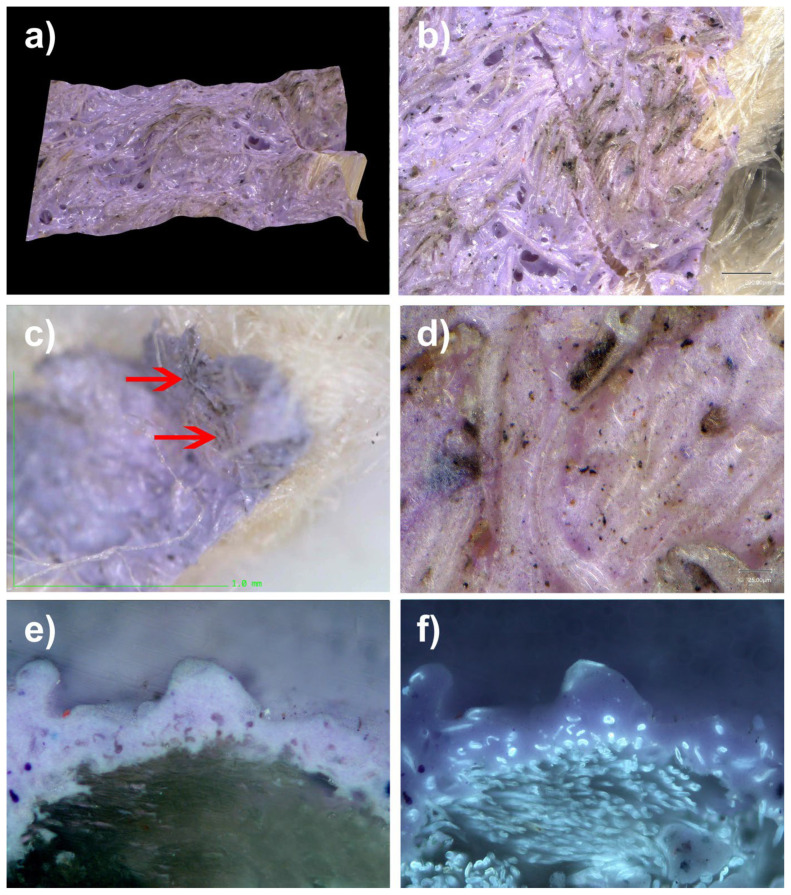
(**a**) View of a sample under an ultra-high-accuracy 4 K microscope: mauve area soiled by dust and dirt. Magnification, 150×. (**b**) Ultra-high-accuracy 4 K microscope view at 20 µm scale: anchored dirt is clearly visible. (**c**) Dirty area in the cross-section. Magnification, 200×. (**d**) Ultra-high-accuracy 4 K microscope view at 25 µm scale: dirt and particulate matter are clearly caught in the paint layer. Magnification, 1000×. (**e**) Enlarged observation of film areas penetrated by grains of dust (Lens: MPlan 20×/0.45). (**f**) Enlarged observation of film areas penetrated by grains of dust (Lens: MPlan 20×/0.45) under UV lighting.

Examination of a paint layer created at Magna de Bocour, directly applied to the canvas, without any ground layer underneath [98] (see Figure 20), during restoration work enabled us to examine areas of trapped blackish dirt on the surface of the canvas grain (Figure 20).

The obvious anchoring of the nanometric dirt seemed to indicate the deep penetration of the pictorial layer, applied in a solvent phase but using a resinous binder with which the Tg is equipped, at a temperature of 20 °C, according to the manufacturer’s technical sheets. Acryloid F-10 is manufactured by Rohm and Haas and is a resin based on an n-butyl methacrylate copolymer. It is the softest and most elastic of the acryloids. It is most often blended with Acryloid B-67 to produce picture varnishes. Acryloid F-10 is only supplied as a 40% solid in mineral thinner. Acryloid F-10 produces a clear film with an ultimate hardness of 2–3 KHN and a glass transition temperature of 20 (Tg °C) [99].

**Figure 21:**
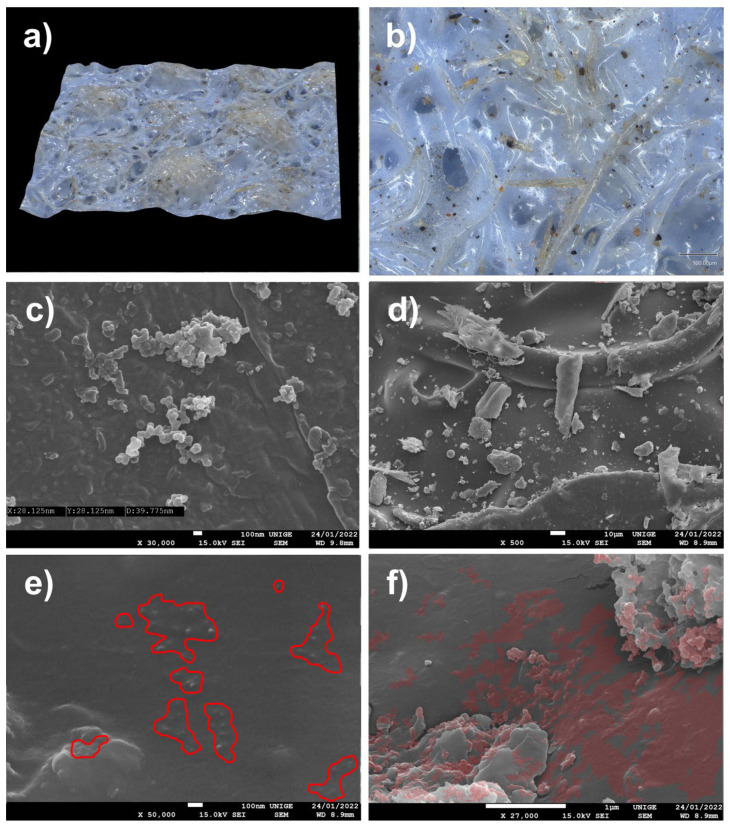
(**a**) View under an ultra-high-accuracy 4 K microscope of a sample of phthalocyanine blue from a Magna paint dating from 1973: principally, the crest of the canvas is the dirtiest area. Magnification, 100×. (**b**) View of the sample under an ultra-high-accuracy 4 K microscope. Magnification, 300×. (**c**) Measurement by SEM of particulate matter trapped in the paint layer. Magnification, 30,000× at 100 nm scale; the average diameter of the particle is around 40 nm. (**d**) View of the sample with SEM: numerous particles clearly have different sizes, but all are firmly fixed. Magnification, 500×. (**e**) Agglomerates of nanoparticles deeply trapped in the acrylic surface, surrounded in red in the SEM picture, at a magnification of 50,000×. (**f**) Extension of nanoparticles trapped in the acrylic surface, identified in red in the SEM picture. Magnification, 27,000×.

The similarity of the facies of the anchors with acrylic paints in emulsion is quite interesting in the context of this study; we were able to take a micro-sample from the back of the work in the area of the painted ties (crosspieces) (Figure 21).

The cross-section indicated the penetration of the microparticles into the paint material; it is also interesting to see the paint layer being absorbed directly into the canvas, following its structure (see Figure 20).

#### 8.10.2. Old Packing Sheet from an Artwork by Kenneth Noland

**Figure 22:**
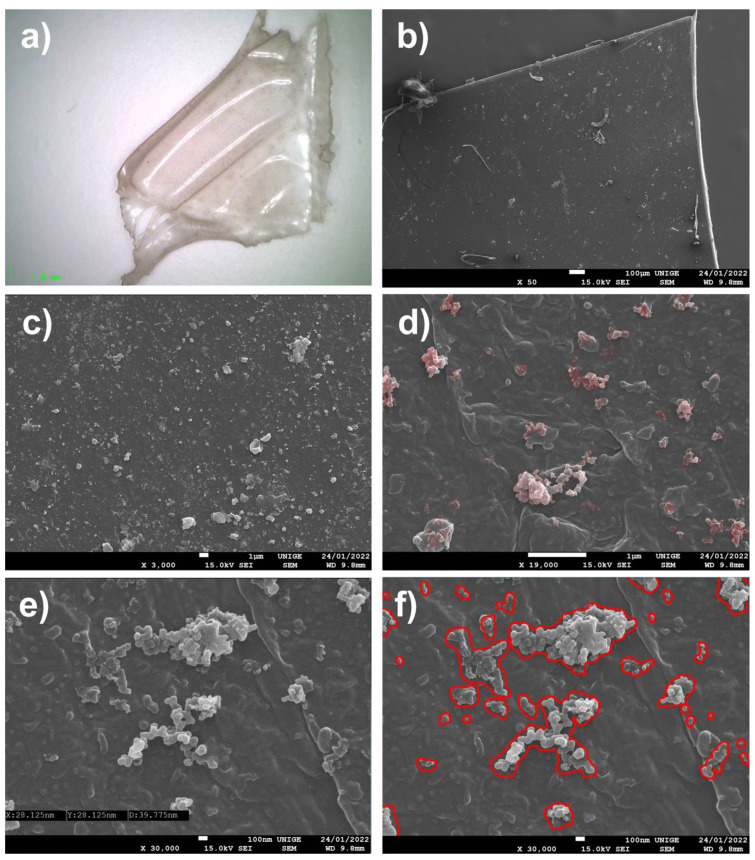
(**a**) Sample of polyethylene film view under a digital microscope. Magnification, 20×. (**b**) Sample of polyethylene film viewed under SEM. Magnification, 50×. (**c**) The abundance of particulate matter of different sizes and nanoparticles, deposited on the film and fixed firmly. Magnification, 3000×. (**d**) Extension of nanoparticles fixed on the surface, identified in red in the SEM picture. Magnification, 19,000×. (**e**) Measurements by SEM of the nanoparticles. Magnification, 30,000× at 100 nm scale; average diameters of around 40 nm. (**f**) Aggregates of nanoparticles on the surface, surrounded in red in the SEM picture. Magnification, 30,000×.

The back of the canvas also revealed old polyethylene packaging (perhaps from the 1970s). The interest of this period packaging, which was in direct contact with the artwork, lies in the fact that it contains “historical” dust and particulate matter, archiving, in a way, all the layers of dust that have collected for many years (Figure 22).

#### 8.10.3. Observations of Particulate Matter on the Packing

The results of our observations revealed particulates of PM 10 and PM 2.5, perfectly visible nanoparticles that partially covered the packaging film, forming chains of aggregates assembled with strong covalent bonds, although they were ready to migrate mechanically or by static electricity. The measurements carried out with SEM confirmed average PM dimensions of 45 to 95 nm.

#### 8.10.4. Observation of the Paint Layer

SEM examination of the actual paint layer showed particularly significant results in terms of trapping dust particles of PM 10, PM 2.5, and nanoparticles, which penetrated the surface even more deeply than on the investigation mock-ups in some places. Some stickiness was almost completely below the surface and only the tops of the nanoparticles remained sticking out from the surface.

### 8.11. Bertrand Lavier: Untitled (from the Mirror Series) Clear Medium on an Acrylic Mirror, Contained in the Original Artist’s Frame: (61 cm × 61 cm)

#### 8.11.1. Description

**Figure 23:**
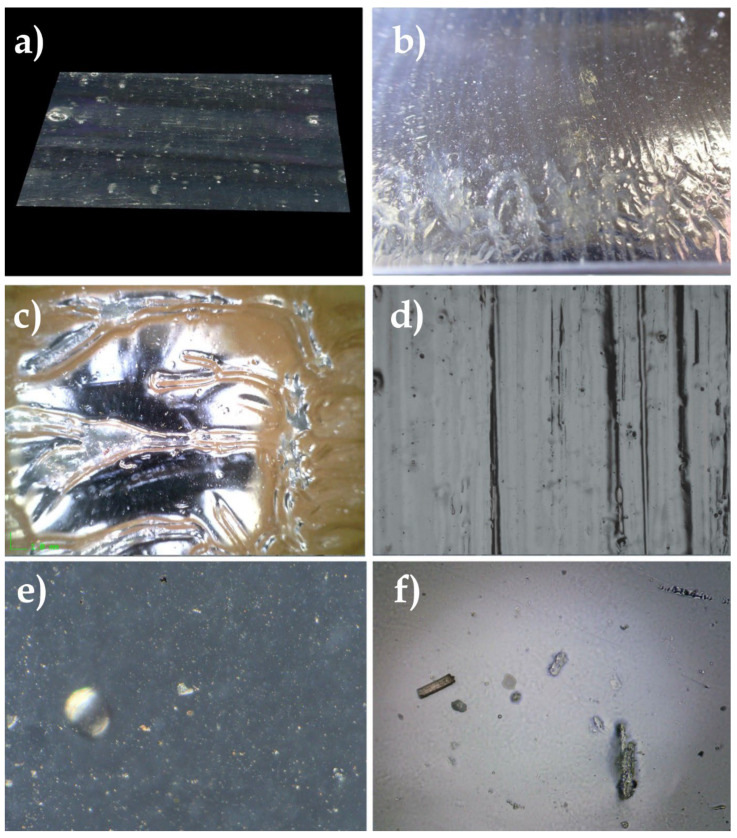
(**a**) Three-dimensional view of the transparent acrylic paint layer under an ultra-high-accuracy 4 K microscope, at a magnification scale of 2000 µm. Magnification, 30×. (**b**) Typical impasto by Lavier: thick transparent touches, close to the original. (**c**) Typical impasto by Lavier: thick transparent touches, close to the original view, under an ultra-high-accuracy 4 K microscope. Magnification of 20× under a digital microscope. (**d**) The brushed paint layer, viewed with an ultra-high-accuracy 4 K microscope. (**e**) Inspection of the paint surface details and trapped particulate matter (PM) at the moment of drying of the acrylic gel. (**f**) Inspection of the surface, with PM and UFP trapped in the acrylic paint, seen under a digital microscope, magnification 730×.

This piece by Bertrand Lavier [100,101] comprises a transparent paint surface made of colorless and glossy acrylic gel layers, directly affixed to a Plexiglas or acrylic resin mirror. Its examination enabled us to identify areas of dust and the trapping of microparticles and nanoparticles, some of which may be constitutive, whereas others will be due to the accumulation and penetration of the acrylic film (Figure 23).

#### 8.11.2. Observations

Gels and mediums can be considered to be colorless paints because they are composed of the same polymers as acrylic paints.

Acrylic heavy or extra heavy gels are based on short rheology. Highly solid gels have higher levels of polymer solids than other gels (approximately 60%, versus 45–50%). In terms of drying, these gels typically take much longer than paints, sometimes taking months to dry thoroughly. Without precautions at the level of the support, they may be liable to discoloration, i.e., support-induced discoloration (SID) [102], which rapidly becomes more visible because of the gel’s transparency and considerable thickness [103].

### 8.12. Deposition of Dust on the Surface of Mock-Up Transparent Acrylic Gel

#### 8.12.1. Mock-Up

Sieved dust was deposited on a mock-up transparent acrylic gel Golden Gel Mediums, Heavy Gel gloss #3050-5, applied to an acrylic mirror, placed in moderate-temperature conditions for a few weeks.

The surface dust was then removed according to the same protocol, in order to examine the presence and penetration of microparticles in the form of PM or UFP.

#### 8.12.2. Observations

Observation of the SEM images made it possible to visualize the topography of the acrylic pictorial surface, where the original aspect is relatively homogeneous according to the magnification scale; however, where certain zones exhibited the trapping of nanoparticles, the microparticles and UFP, in the shape of micro-grains, were partially embedded in the thermoplastic material.

**Figure 24:**
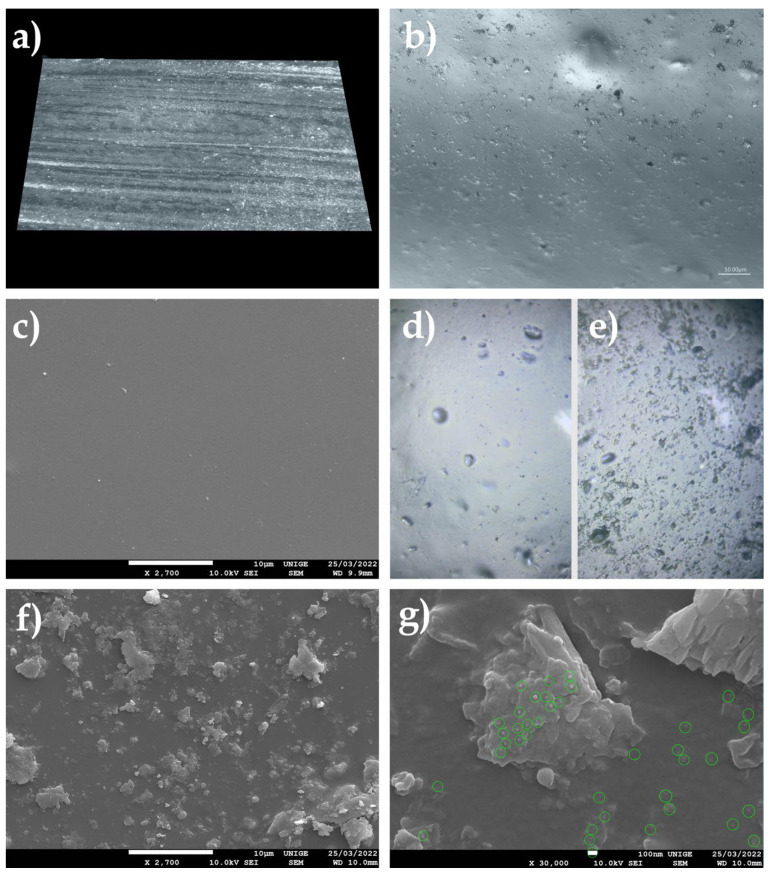
(**a**) Three-dimensional sample of the acrylic mock-up, cleaned after the deposition of dust on the surface, viewed under an ultra-high-accuracy 4 K microscope. Magnification, 30×. (**b**) Acrylic mock-up viewed under an ultra-high-accuracy 4 K microscope: there is visible anchoring of the dust deposited on the surface, at a magnification scale of 50 µm. (**c**) Sample of the acrylic gel layer without dust via SEM, at a magnification of 2700×. (**d**) Sample of the acrylic gel layer without dust via SEM, at a magnification of 730×. (**e**) Sample of the acrylic gel layer with dust, viewed under a digital microscope, at a magnification of 730×. (**f**) PM agglomerates and UFP aggregates trapped in the acrylic surface, viewed with SEM, at a magnification of 2700×. (**g**) View via SEM of PM nanoparticles trapped in the acrylic surface, at a magnification of 30,000×: identification of some UFP indicated by the green circles (at a scale of 100 nm).

Observations performed under a digital microscope demonstrated the possibility of the gradual trapping of unpigmented and uncharged acrylic gel. This can cause visual changes that gradually increase over time, depending on the storage and exposure conditions (Figure 24).

### 8.13. Deposition of PM and UFP Soot on Acrylic Paint on Canvas (1970)

#### 8.13.1. Sample of a Highly Smoke-Treated Paint Layer after a Fire

Examination of the pictorial layer revealed a strong surface of smoke on this acrylic paint. The appearance and difficulty of cleaning soot dust that appeared to be superficial led to some suspicion regarding its penetration of the dermis of the pictorial layer.

#### 8.13.2. Observations

Observation of the SEM images revealed the topography of the acrylic pictorial surface, where the original aspect was relatively homogeneous, according to the magnification scale. Nevertheless, certain zones exhibited trapping of the nanoparticles, the microparticles, and UFP, in the shape of micro-grains that were partially embedded in the thermoplastic material.

High-resolution 4 K microscopic examinations illustrated the nature of the PM soot and UFP soot that were trapped, accompanied by a few crystallized salts.

**Figure 25:**
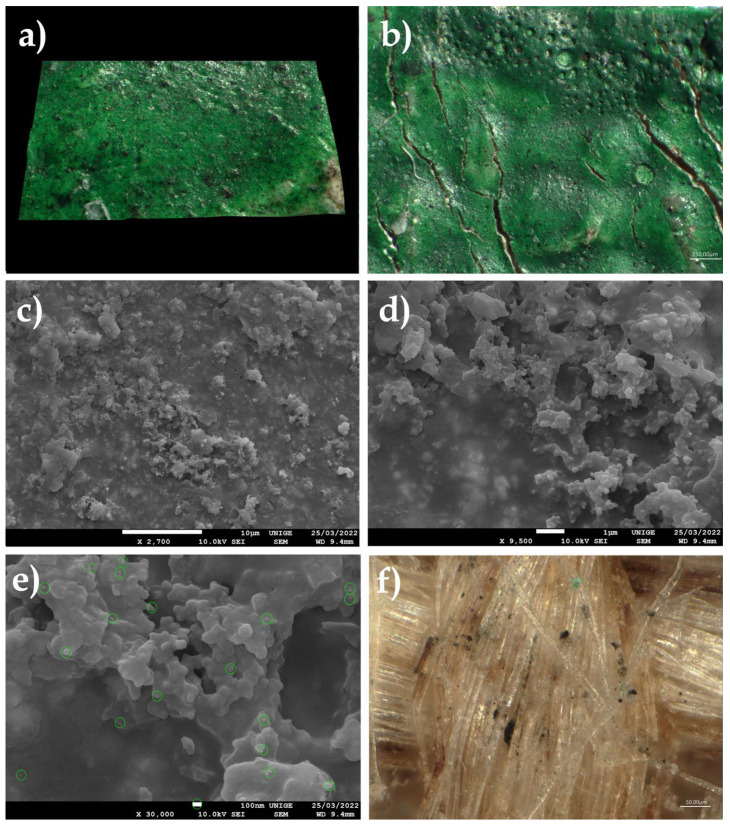
(**a**) View of a sample of acrylic paint from the 1970s, seen under an ultra-high-accuracy 4 K microscope, at a magnification of 500×: we can see the penetration of dust and particles of soot. (**b**) View of the anchoring of soot deposited on the surface under an ultra-high-accuracy 4 K microscope, at a magnification of 100×. (**c**) View of the sample via SEM, at a magnification of 2700×: abundant PMs are firmly anchored in the layer. (**d**) View of the sample via SEM, at a magnification of 9500×: PM and UFP are present in the layer. (**e**) View of the aggregates of PM and UFP trapped in the acrylic surface, viewed using SEM, at a magnification of 30,000×: the identification of some UFP with green circles, seen at a scale of 100 nm. (**f**) The reverse of the canvas, viewed under an ultra-high-accuracy 4 K microscope, at a magnification scale of 50 µm. The PM and UFP of soot have even penetrated fibers in the weft.

The back of the canvas, which did not appear to have been impacted by accidental smoking, in fact showed the penetration of PM and UFP soot, trapped in the fibers of the weft itself (Figure 25).

#### 8.13.3. Collecting PM and UFP

The facies of the soot could be studied on the different fibers of the absorbent paper and the hydrophilic cotton used for cleaning the surface of the artwork.

**Figure 26:**
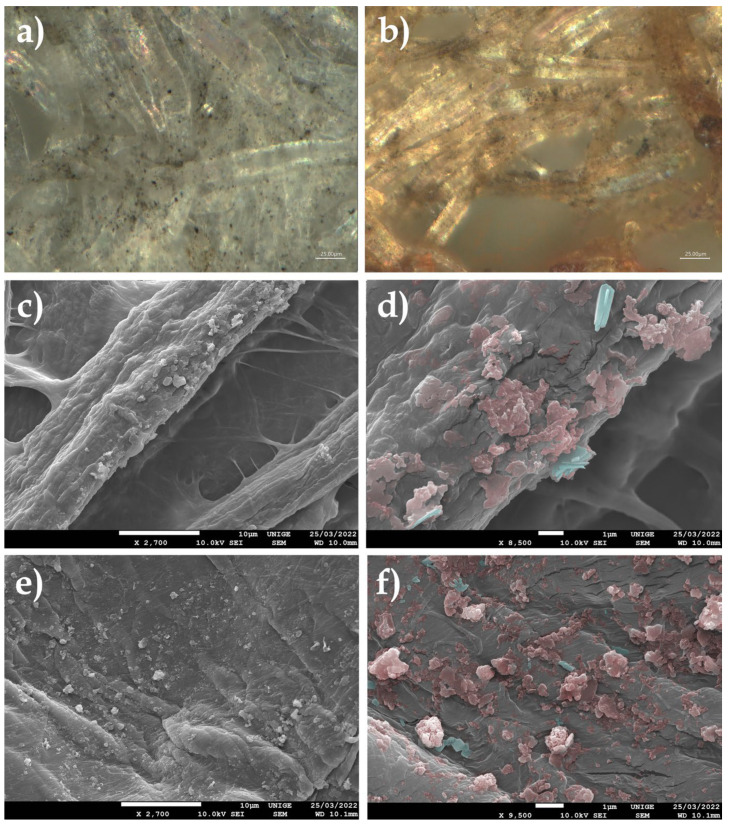
(**a**) Absorbent paper, viewed under an ultra-high-accuracy 4 K microscope, at a magnification of 1000×: dust and soot were collected during cleaning. (**b**) Cotton wool (swab), viewed under an ultra-high-accuracy 4 K microscope, at a magnification of 1000×: dust and soot were collected during cleaning. (**c**) Acid-free tissue paper for paper restoration L3: soot particles aggregated in fibers after cleaning. The observation was viewed under SEM, at a magnification of 2700×. (**d**) Acid-free tissue paper for paper restoration L3: agglomerates of PM and UFP were located in red on the picture taken via SEM (magnification, 8500×); also present are micro-crystals of salt, identified in blue. (**e**) Absorbent paper: aggregates of the particles of soot in the fibers after cleaning: observed via SEM (magnification, 2700×). (**f**) Absorbent paper: agglomerates of PM and UFP are identified in red on the picture taken via SEM (magnification, 9500×). Micro-crystals of salt are identified in blue.

The soot particles were clearly aggregated in the fibers of the non-acid and absorbent papers (Figure 26).

### 8.14. Deposition of Dust on the Surface of the Gloves Used for Handling Artworks

#### 8.14.1. Description

The commonplace contemporary use of protective gloves for handling artworks may cause unexpected damage to acrylic or pressure-sensitive surfaces.

Although it is true that hands covered by gloves effectively protect surfaces from organic deposits or dirt and fingerprints, the poor management of these protection measures will cause damage and encourage the accumulation of dirt on surfaces when the gloves are dirty or are simply worn for too long.

This aspect of the study seeks to contribute to understanding these very real phenomena resulting from collateral soiling due to the simple handling of artworks.

The three most common types of protective glove materials are cotton, PVC pimples, and the most commonly used protective gloves: polyester/nylon gloves, backed with nitrile foam on the outside.

An observation of the parts in contact (on the internal faces of the glove) is indicative of the active trapping of microparticles, accompanied by UFP.

**Figure 27:**
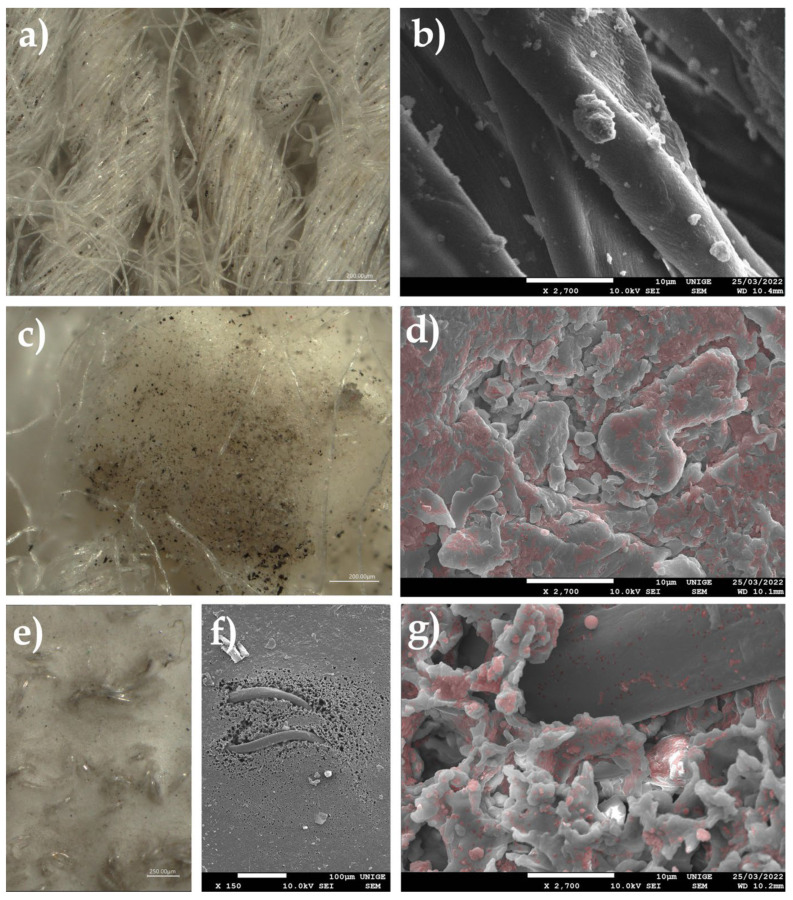
(**a**) Detail of a cotton glove viewed under an ultra-high-accuracy 4 K microscope, at a magnification of 200 µm: trapped dust and dirt was collected during the handling of artworks. (**b**) View via SEM (magnification 2700×) of fibers from the cotton jersey: PM and UFP are hooked on each fiber. (**c**) Detail of a dot from an anti-slip dotted glove, viewed under an ultra-high-accuracy 4 K microscope, magnification scale of 200 µm: dust and dirt collected during the handling of artworks. (**d**) View under SEM of a dot from an anti-slip glove: PM and UFP (identified in red) are hooked in the wears and hollows of the PVC, at a magnification of 2700×. (**e**) Detail of the relief of a nitrile glove, viewed with an ultra-high-accuracy 4 K microscope, at a magnification scale of 250 µm: the trapping of dust and dirt was collected during the handling of artworks. (**f**) Relief of a nitrile glove under SEM, at a magnification of 150×: the skin of the nitrile foam degrades mechanically and traps PM and UFP. (**g**) View of PM and UFP trapped in closed-cell rubber foam (identified in red), opened because of abrasion of the skin at the surface, viewed under SEM at a magnification of 2700×.

- The jersey weave of bleached cotton gloves very easily trap PM, which inevitably comes in contact with the artwork during handling (Figure 27a,b).
- The dots of pimple-palm gloves are often made of PVC rubber; they wear out and catch dust, while quickly accumulating dirt: unfortunately, this creates a rubber-stamp effect on the pictorial surfaces, due to the transfer of dirt from the gloves (Figure 27c,d).
- The surfaces of polyester/nylon gloves backed with rubber nitrile foam commonly have a certain structure that reinforces their non-slip characteristics. Observation via SEM allowed the discovery that it is mainly at the level of these reinforcements that the skin of nitrile foam degrades mechanically, trapping PM and UFP in the cavities of closed-cell rubber foam, which can then be redeposited on the paint surfaces (Figure 27e–g).

#### 8.14.2. Mock-Up

**Figure 28:**
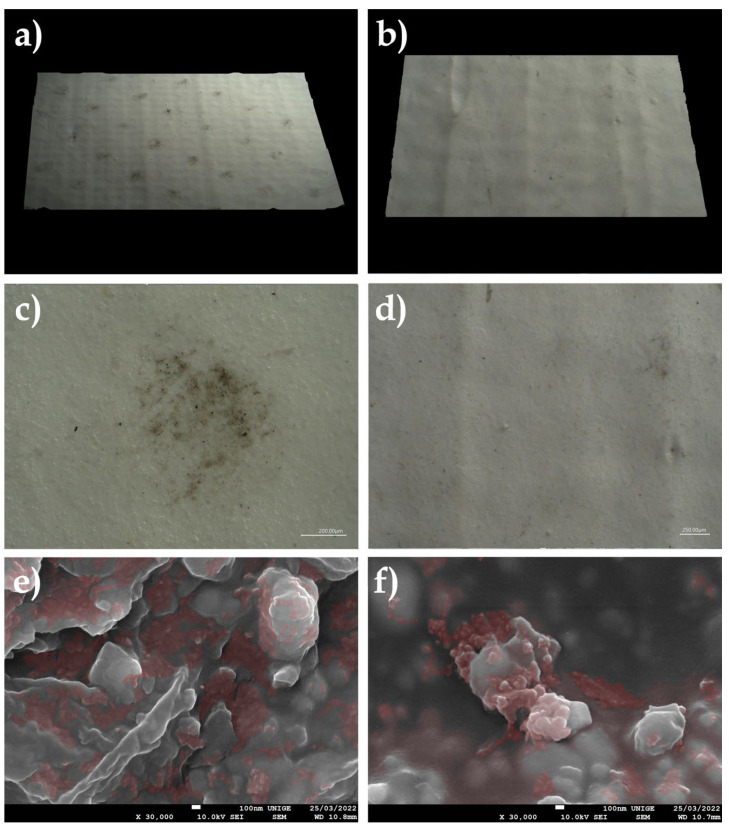
(**a**) Three-dimensional sample of an acrylic mock-up with the deposition of dots; the traces come from an anti-slip glove, viewed under an ultra-high-accuracy 4 K microscope, at a magnification of 20×. (**b**) Three-dimensional sample of the acrylic mock-up with the deposited dust on the surface, viewed under an ultra-high-accuracy 4 K microscope at a magnification of 50×. (**c**) View via an ultra-high-accuracy 4 K microscope (magnification, 200×) of the acrylic mock-up, with a dot mark deposited on the surface. (**d**) View of the acrylic mock-up with deposited dust on the surface, viewed under an ultra-high-accuracy 4 K microscope, at a magnification of 100×. (**e**) Aggregates of PM and UFP trapped in the hollows of the acrylic paint mock-up, at an SEM magnification of 3000×. These are identified in red at a scale of 100 nm. (**f**) View via SEM of the aggregates of PM and UFP trapped in the hollows and the paint surface of the acrylic mock-up. These are identified in red at a scale of 100 nm and a magnification of 30,000×.

Impressions made with pimple-palm gloves and polyester/nylon gloves backed with nitrile foam were voluntarily deposited by simple contact on the mock-up surfaces, made of a white acrylic pictorial layer (titanium white n° 191, Lascaux Artist©, Brüttisellem, Switzerland) (Figure 28a–d).

#### 8.14.3. Observations

Observation with a 4 K high-resolution digital microscope and via SEM made it possible to identify and locate the emissions of PM and UFP in the dermis of the acrylic pictorial layers of the mock-up (Figure 28e,f).

## 9. Conclusions

In the current circumstances, a conclusion seems difficult to draw; considering nanoparticles as a nano-problem denies their impact in the field of artwork conservation, above all, when they are only viewed as a technological improvement. Because of their imperceptible nature, their effects are insidious but real and, ultimately, can have irreversible consequences. The various documents illustrating our study testify to this finding, as does our professional experience.

If the inhibition of airborne nanoparticle production is unrealistic, it would be wise, even before tackling the subject of cleaning pictorial layers that are soiled by these stains (which are, unfortunately, often irreversible), it seems best to consider preventive solutions, particularly with regard to the storage of works of art in the repositories of museums and public collections, in order to reduce the phenomenon of nano-dusting as much as possible.

The careful choice of wrapping or packing materials should be made among materials with low levels of charge accumulation and electrification by rubbing (triboelectricity) [104], along with the most conductive properties possible (in order to almost immediately dispel the static electricity) [105,106,107].

It is also necessary to avoid the wrapping making contact with the surface of the artwork as much as possible, whatever the quality of the sheeting used.

The installation of an antistatic carpet at the entrance of museums’ storage facilities and the use of protective antistatic footwear made of micro-porous fabric (ibid. [105]) may be useful. In the same way, earthing metal objects (such as shelves, furniture, and racks) might be an appropriate solution. This is a preventive alternative measure, in order to avoid electrostatic risk and the possibility of electric shocks by connecting all surrounding metallic objects (ibid. [105]).

There are also currently fine-particle-trapping systems that are used in the medical and IT fields, which function as electrostatic precipitators, along with HEPA-type mechanical purifiers.

The problem of particulates must be considered at all levels, well before deep and probably indelible stains on painted layers are noticed once it is too late. It seems rational to inform museums and galleries of the importance of dusting management for artworks composed of polymers that are sensitive to thermal variations, keeping Tg values close to exhibition or storage temperatures.

Preventive guidelines for these artworks should be particularly strict when gloves are employed or during their handling and packing, as these could be sources of nanoparticles, thus causing potentially irreversible damage.

Regarding nanoparticles, UFP, and particulate matter, prevention is essential in this context, and it must also be included in exhibition instructions for these artworks, which can thereby suffer from large thermo-mechanical amplitudes, the consequences of which include an increase in the movement of the polymer films, and, hence, the penetration of dust and thin and ultra-thin particles.

Whether we are concerned with artworks or health, we must be attentive and cautious about the harmful effects of UFPs, which current filtration methods have not yet rendered negligible.

## Figures and Tables

**Table 1:** Examples of various physico-chemical elements after EDS analysis on the dust collected on the back of an artwork.

EDS Energy-Dispersive X-ray Spectroscopy ZAF Method Standardless Quantitative Analysis													
Element	C	N	O	Na	Mg	Al	Si	S	Cl	K	Ca	Fe	Total
Analysis I 1/4	30.28	14.7	28.47	0.68		3.31	5.93	3.15		1.64	10.9	0.94	100.00
Analysis I 2/4	26.09		31.07			6.62	8.94	3.35		4.24	17.59	2.11	100.00
Analysis I 3/4	22.85		45.09	0.82		1.59	2.72	1.72		1.56	22.09	1.56	100.00
Analysis I 4/4	29.42		44.69	0.39	0.37	1.22	2.1	3.03		0.64	16.57	1.57	100.00
Analysis II 1/1	25.32		44.05				0.71	10.88		10.11	8.93		100.00
Analysis III 2/4	19.84		34.51	1.21	5.25			13.66		11.02	14.51		100.00
Analysis III 3/4	46.17		38.75					3.1		0.81	11.16		100.00
Analysis III 4/4	55.61		37.11	0.49				0.69	0.57	0.65	4.88		100.00

## Data Availability

Not applicable.
